# Highly active repeat-mediated recombination in the mitogenome of the aquatic grass *Hygroryza aristata*

**DOI:** 10.1186/s12870-024-05331-x

**Published:** 2024-07-08

**Authors:** Huijun Wang, Zhigang Wu, Tao Li, Jindong Zhao

**Affiliations:** 1 Institute of Hydrobiology, Chinese Academy of Sciences, Wuhan, China; 2 University of Chinese Academy of Sciences, Beijing, China; 3 State Key Laboratory of Protein and Plant Genetic Engineering, College of Life Sciences, Peking University, Beijing, China

**Keywords:** *Hygroryza aristata*, Mitochondrial genome, Repeat, Homologous recombination, Intracelluar gene transfer

## Abstract

**Background:**

Floating bamboo (*Hygroryza aristata*) is an endangered species with a narrow native distribution and is renowned for its unique aesthetic qualities, which holds significant ecological and ornamental value. However, the lack of genetic information research, with only one complete plastome available, significantly hampers conservation efforts and further research for this species.

**Results:**

In this research, we sequenced and assembled the organelle genomes of floating bamboo, including the mitogenome (587,847 bp) and plastome (135,675 bp). The mitogenome can recombine into various configurations, which are mediated by 25 repeat pairs (13 SRs, 6 MRs, 1 LR, and 5 CRs). LR1 and SR5 are particularly notable as they have the ability to combine with other contigs, forming complex repeat units that facilitate further homologous recombination. The rate of homologous recombination varies significantly among species, yet there is still a pronounced positive correlation observed between the length of these repeat pairs and the rate of recombination they mediate. The mitogenome integrates seven intact protein-coding genes from the chloroplast. The codon usage patterns in both organelles are similar, with a noticeable bias towards C and T on the third codon. The gene map of Poales shows the entire loss of *rpl6*, succinate dehydrogenase subunits (*sdh3* and *sdh4*). Additionally, the BOP clade retained more variable genes compared to the PACMAD clade.

**Conclusions:**

We provided a high-quality and well-annotated mitogenome for floating bamboo and demonstrated the presence of diverse configurations. Our study has revealed the correlation between repeat length and their corresponding recombination rate despite variations among species. Although the mitogenome can potentially exist in the form of a unicircular in vivo, this occurrence is rare and may not be stable.

**Supplementary Information:**

The online version contains supplementary material available at 10.1186/s12870-024-05331-x.

## Backgroud

*Hygroryza aristata*, also known as floating bamboo, is an aquatic, perennial grass renowned for its ability to float on the surface of slowly flowing freshwater bodies due to its spongy, inflated leaf sheaths [1, 2]. *H. aristata* is native only to the tropical regions of Southeast Asia [1, 2]. However, due to the limited native distribution range, habitat destruction and fragmentation, as well as extensive use as feed for fish and livestock [3, 4], the rarity of this species is increasing rapidly [5]. In 2021, it was classified as a second-level nationally protected wild plant in China. Meanwhile, owing to its unique aesthetic qualities, this species is widely favored by aquarium enthusiasts worldwide, posing some invasive risks outside of its native range. Increasing concerns about water pollution in recent years have brought unprecedented attention to *H. aristata* as a natural material for water purification [6, 7]. Additionally, it serves as a source of chemical compounds, such as lignan and indole alkaloids, which are utilized in traditional medicine for their anti-inflammatory and antioxidant properties [8]. However, research on its genetic information is severely lacking. Currently, there is only one complete chloroplast record of *H. aristata* available [9], severely hampering conservation and research efforts for this species. Poaceae is the fifth-largest family in angiosperm (~ 12,000 species in 12 subfamilies) and holds the most significant economic importance for humans [10]. It includes essential food sources such as rice, maize, wheat, as well as numerous forage and biofuel species. Members of the Oryzoideae subfamily (such as *Oryza* and *Hygroryza*) are more adapted to aquatic habitats, unlike most other grasses that thrive in dry ecosystems [11].

Organelles believed to have originated from ancient endosymbiotic events are present in most eukaryotes [12, 13]. These organelles retain their genomes which originated independently from nuclear genomes. However, only a limited set of genes remains in these genomes to maintain primary biogenesis during long-term symbiosis [12]. Nonetheless, the biogenesis of these organelles also relies on the coordinated expression of genes in the nucleus, rendering them semiautonomous. Plants possess two organelles: the chloroplast (cp) and the mitochondrion (mt). Chloroplast can be considered the cornerstone of ecosystems as an essential organelle for carbon fixation via photosynthesis. Studies have confirmed that plant mitochondria are crucial for respiration, metabolism, programmed cell death (similar to animal mitochondria) and play a significant role in cytoplasmic male sterility breeding [14–16], making them an indispensable component in breeding research.

As of January 2024, NCBI has published complete plastomes of 6,116 land plants, while the number of complete mitogenomes is only 272 (this count is slightly lower as it includes only mitogenomes with a single chromosomal structure; Table 1). This stark contrast is attributed to the complexity of the mitogenome in embryophyta. In comparison to the relatively compact and conserved genomes of plant chloroplasts and animal mitochondria, plant mitogenomes exhibit unique features, including extensive variation in size (Table 1), frequent genome rearrangements, the incorporation of DNA from various sources through intracellular and horizontal transfer, and extensive RNA editing sites [17–19]. The variability of plant mitochondrial genomes can even fluctuate significantly within a single species [20, 21]. Among the major branches of eukaryotes, embryophyta mitochondria have the largest genome size and the highest GC content (~ 44.16%; Table 1). These distinctive features among plant mitogenomes may elucidate why so few have been appropriately assembled and characterized. However, despite the structural variability of plant mitochondria, the coding sequences within their genomes are highly conserved in terms of both number and genetics [12, 22].

**Table 1:** Summary of the complete organelle genomes with a single chromosomal structure published on NCBI

Organelles	Classes	Number of unique species	Average size (kb)	Range of size (kb)	Average GC content (%)
**mt**	Animals	10,845	16.40	8.12-48.16	36.15
	Fungi	499	66.66	12.06-332.17	27.08
	Green Algae	84	57.32	13.00-241.74	37.75
	Land Plants	272	404.02	99.86-1999.60	44.16
	Protists	364	34.77	5.80-119.31	29.67
**cp**	Green Algae	150	160.07	48.19-1352.31	32.68
	Land Plants	6,116	152.61	15.55-242.58	37.40
	Protists	245	148.25	33.54-610.06	30.52

Note: accessed on January 19th, 2024, https://ftp.ncbi.nlm.nih.gov/genomes/refseq/

Although most mitogenomes can be mapped as a unicircular structure on physical maps, referred to as the “master circle” model, more experimental evidence has confirmed that this is not the dominant structure of mitochondria in vivo [22, 23]. Instead, they are constantly involved in dynamic homologous recombination (HR) mediated by repeat pairs, resulting in a dynamic mixture of small-sized forms, including branched linear, circular, linear, degraded, comet, and branched circular structures, among others [23, 24]. HR is an important and evolutionarily conserved homology-dependent DNA repair process used to eliminate potentially harmful lesions, particularly double-stranded breaks (DSBs) [18]. HR also exists in the plastome, albeit at a lower frequency. Fluorescence in situ hybridization (FISH) shows that reorganized cpDNA represent between 0.8% and 2% of all [25]. Most published plant mitogenomes contain at least one pair of repeats that can serve as sites for inter- or intramolecular recombination, resulting in multiple alternative arrangements (isoforms). For now, the only exception is *Brassica hirta*; restriction mapping studies have revealed that, unlike other cogeneric species, the mitochondrion of *B. hirta* exists in vivo as a slightly smaller single circular chromosome [26]. It has been proposed that the large repeats can frequently recombine intra- or intermolecularly, while the small repeats may lead to subtle rearrangements [18]. However, quantitative studies based on long sequencing reads show significant variations among species [27–36].

In this research, we successfully: (1) utilized tissue culture techniques for the conservation of floating bamboo germplasm resources; (2) sequenced and assembled the mitogenome of floating bamboo; (3) verified the positive correlation between the length of repeat sequences and the mitogenome recombination rate; (4) further investigated sequence transfer between mitochondrion and chloroplast, revealing similarities in codon usage bias between these two organelles; (5) explored gene loss pattern in the mitogenome of Poales.

## Methods

### Plant material and genome sequencing

The sample of *Hygroryza aristata* was collected from Wuhan, China and preserved at the Institute of Hydrobiology, Chinese Academy of Sciences through tissue culture (Fig. S1). Genomic DNA was extracted from fresh leaves using a modified CTAB method [37]. The purified DNA was used to prepare libraries for both Illumina (VAHTS Universal Plus DNA Library Prep Kit, Vazyme Biotech, Nanjing, China) and Nanopore (SQK-LSK110 Ligation Sequencing Kit, Oxford Nanopore Technologies, Oxford, UK) following the manufacturers’ protocols. The Illumina library was sequenced on the Illumina Hiseq4000 platform (Illumina, San Diego, USA). Raw reads were filtered by Fastp v0.23.2 [38] with the parameters “qualified_quality_phred 20; --unqualified_percent_limit 50; --length_required 35; --detect adapter_for_pe”. The Nanopore library was sequenced on the PromethION P48 sequencer (Oxford Nanopore Technologies, Oxford, UK), base calling and prefiltering were performed by Guppy v6.3.8 (Oxford Nanopore Technologies, Oxford, UK). To ensure that our data were sufficient for the assembly of organelles, we utilized Jellyfish v2.2.10 [39] and GenomeScope v2.0 [40] to assess the whole genome size based on *k*-mer.

### Assembly and annotation of organelle genomes

The chloroplast genome was assembled using GetOrangelle v1.7.7.0 [41] with Illumina whole-genome sequencing (WGS) data as input.

To detect repeat sequences in the mitogenome of *H. aristata* that could potentially facilitate recombination and provide insights into its variable conformations within the organism, we employed a sophisticated assembly strategy. 1). We utilized both Flye v2.9.3 [42] and Unicycler v0.5.0 [43] with default parameters to initially assemble the Nanopore long reads. 2). All contigs were searched against the complete set of mitochondrial protein-coding genes encoded by angiosperm using BLASTn v2.13.0+ [44] with an e-value cutoff of 1e-5. 3). Then, we used 44 candidate contigs as references to identify potential mitochondrial reads from the Illumina WGS data using BWA-MEM2 v2.2.1 [45]. 4). Following this, a *de novo* assembly was performed on the extracted Illumina reads using SPAdes v3.15.5 (*k*-mer = 27, 53, 71, 87, 99, 111, 119, 127) [46]. 5). The assembled graph was then simplified by removing edges with less than 10× coverage depth. Additionally, chloroplast- and nuclear-derived nodes were manually removed using the interactive visualization interface of Bandage v0.8.1 [47]. During this process, we detected several repeats that potentially mediated HR, as they exhibited multiple connections and doubled coverage depth. 6). Finally, with the assistance of Nanopore long reads, we successfully obtained the complete mitogenome of *H. aristata* as a single circular molecule (i.e., the “master circle” model).

The annotation for the cp and mt genomes was conducted using GeSeq [48], with reference to the previously released cp genome of *H. aristata* (NCBI accession number NC_058302.1) and the available mitogenomes of Poales (Table S1), respectively. Additionally, tRNAs were validated by tRNAscan-SE with default settings [49]. The PCGs underwent manual verification and editing using Geneious Prime R9.0.2 [50]. Finally, both circular organelles were visualized using OGDRAW [51].

### Detection of genome recombination

During the process of assembling the mitogenome, we detected 25 repeat sequences that have the potential to facilitate HR. Although a “master circle” mitogenome was obtained with the assistance of Nanopore long reads, it is essential to acknowledge that this assembly merely represents one possible configuration. To confirm the occurrence of HR and to evaluate the correlation between the length of repeat sequences and their associated recombination frequency, Nanopore long reads were mapped to these presumed conformations. Each repeat had two paths indicating the primary conformation (m1 and m2) and two paths representing the secondary conformation (s1 and s2). Utilizing minimap2 v2.26 [52], Nanopore long reads were individually aligned to these four hypothetical conformations. Each hypothetical path included the repeat sequence and its neighboring contigs. If the adjacent contig was shorter than 1 kb, an additional contig was included to ensure alignment accuracy. Only reads that aligned with the entire repeat sequence and also included regions extending at least 100 bp on both flanks of the repeat sequence were deemed supportive of that specific configuration. If two paths supported the same conformation (m1 and m2, s1 and s2), only the path with the highest count was acknowledged. Recombination frequency of m1/m2 (F_m_) is calculated as max(m1|m2). Similarly, the recombination frequency of s1/s2 (F_s_) is calculated as max(s1|s2). The recombination rate is determined by min(F_m_|F_s_)/(F_m_+F_s_). Therefore, according to this calculation method, the recombination rate should range from 0 to 50%. Meanwhile, we have included ten other angiosperm species that have been subject to qualitative assessment of recombination frequency in prior studies [27–36] while investigating the inter-species variations in repeat sequences.

### Detection of intracellular gene transfer

Currently, only the two organelle genomes are available for identifying intracellular sequence migration in *H. aristata* due to the lack of a published nuclear genome. To identify homologous sequences that could potentially be transferred among the organelles, we compared the cpDNA and mtDNA using BLASTn with the following parameters: “-evalue 1e-5; -word_size 7; -max_hsps 10”. The identified transferred DNA fragments were extracted and annotated using GeSeq. Subsequently, these results were visualized using Tbtools v2.065 [53].

### Analysis of repeat elements

We analyzed three different types of repeat sequences in both organelles. (1) Simple sequence repeats (SSRs), also known as microsatellites, were identified using MISA [54], with parameter settings of 10, 5, 4, 3, 3, and 3 for mono-, di-, tri-, tetra-, penta-, and hexanucleotide repeats, respectively. (2) An online tool REPuter [55] was utilized to identify four types of dispersed repeats, namely forward (F), palindromic (P), reverse (R), and complement (C) repeats (Hamming Distance = 3; Minimal Repeat Size = 30 bp). (3) Tandem repeat elements were identified using TRF v4.09 [56] (Match = 2; Mismatch = 7; Delta = 7; PM = 80; PI = 10; Minscore = 50; MaxPeriod = 500).

### Codon usage bias

To investigate the bias pattern of codon usage in these two organelles, we extracted a total of 51 cp CDS and 34 mt CDS, following the criteria outlined by Zhang et al. [57]. The basic compositional properties of these genes, including overall GC content, GC1/2/3 (the GC content at the first, second, and third base position of the codons), GC12 (the mean of GC1 and GC2), as well as GC3s (the GC content at the third base position of synonymous codons, excluding Met, Trp, and the three stop codons), were calculated using EMBOSS v6.6.0 [58]. Subsequently, CodonW v1.4.2 [59] was employed to conduct the codon usage analysis, which included calculating the effective number of codons (ENC) and determining the relative synonymous codon usage (RSCU). The ENC value ranges from 20 (extreme bias) to 61 (no bias), indicating the degree of codon usage bias. Typically, a threshold of 35 is used to evaluate the strength of codon preference [60]. Neutrality plot analysis, ENC-plot analysis, and Parity rule 2 (PR) plot analysis were performed following the methods described by Liu et al. (2020) [61].

### Identification of RNA editing sites

To identify RNA editing sites occurring in PCGs, we downloaded a transcriptomic data from NCBI (SRR16192102). We then mapped the RNA-sequencing data to the PCGs extracted from the organelle genomes using BWA-MEM2. Subsequently, we utilized REDItools v2.0 [62] to analyze the base composition and coverage of each site, with the following parameters: --min-read-length 50; --min-read-quality 35. For high-copy chloroplast PCGs, a minimum coverage of 20× and at least 10% read support were required to consider as an RNA editing site. However, for mitochondrial PCGs with low copy number and low expression, the coverage threshold was relaxed to 10×.

### Phylogenetic and synteny analyses

Phylogenetic analyses were conducted using all available mitogenomes of Poales (Table S1). Due to incomplete annotation information in these genomes and the absence of many core genes, we conducted a reannotation of them. Subsequently, we obtained a dataset consisting of 32 genes, including 24 core genes and 8 variable genes (*rps1*, *rps2*, *rps3*, *rps4*, *rps7*, *rps12*, *rps13*, and *rpl16*). Each gene was aligned using MAFFT v7.508 [63] and manually trimmed before being concatenated. We utilized IQ-TREE v2.2.0.3 [64] to conduct phylogenetic analysis, with the TVM + F + I + I + R2 model selected as the best-fit nucleotide substitution model.

Based on the phylogenetic results, we selected two species from *Oryza* (*O. rufipogon* and *O. sativa*) that are most closely related to *H. aristata* to study the structural changes of their mitogenomes. First, the genomes were pairwise compared using the BLASTn program. Then, only homologous regions with a length exceeding 1,000 bp were retained. Finally, the visualization was conducted using RIdeogram [65].

## Results

### Mitochondrial genome assembly and annotation

**Fig. 1:**
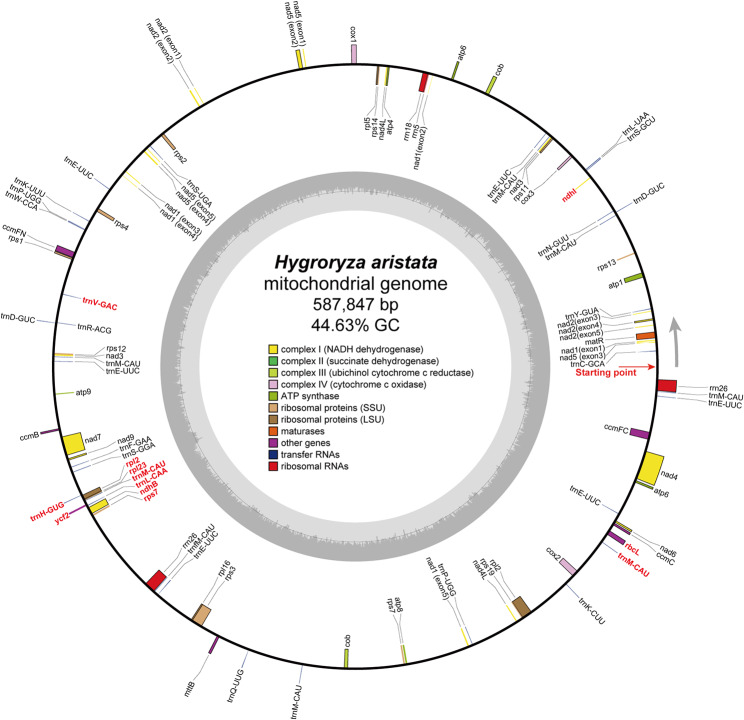
A representative mitochondrial genome map of *Hygroryza aristata*. The outer circle represents gene locations, while the inner circle displays GC content. Genes belonging to different functional groups are color-coded for easy identification. The red arrow indicates the starting point of the mitogenome. Genes transcribed clockwise and counter-clockwise are depicted on the inside and outside of the circles, respectively. Complete gene transfers from chloroplast DNA are highlighted in red

In total, we generated 23.60 Gb Illumina reads and 22.54 Gb Nanopore reads through whole-genome sequencing (Table S2). The whole genome size of floating bamboo was estimated to be around 319.08 Mb (Fig. S2). A subset of 6.43 Gb Illumina short reads was identified as potentially derived from the mitochondrial genome and was subsequently used for *de novo* assembly. Due to the presence of numerous repeat sequences, the initial assembly graph exhibited a complex conformation (Fig. S4). However, leveraging ONT long reads enabled us to represent the mitogenome as a single circular molecule (587,847 bp; Fig. 1), which is significantly larger than the average length of mitogenomes in land plants (404.02 kb; Table 1). The coverage depth of the chloroplast and mitochondrial genomes is 822.3× and 159.5×, respectively (only Illumina reads were considered). The GC content of the mitogenome was 44.63% (Fig. 1), noticeably higher than the GC content of the chloroplast genome of the same species (39.03%; Fig. S3).

In the mitogenome of *H. aristata*, we annotated a complete set of 24 core genes (Fig. 1), including five ATP synthase genes (*atp1*, *atp4*, *atp6*, *atp8*, and *atp9*), nine NADH dehydrogenase genes (*nad1*, *nad2*, *nad3*, *nad4*, *nad4L*, *nad5*, *nad6*, *nad7*, and *nad9*; in addition to a chloroplast-derived *ndhB*), four cytochrome C biogenesis genes (*ccmB*, *ccmC*, *ccmFc*, and *ccmFn*), three cytochrome C oxidase genes (*cox1*, *cox2*, and *cox3*), ubiquinol cytochrome c reductase (*cob*), a transport membrane protein (*mttB*), and a maturase (*matR*). The variable genes in the mitogenome include three large subunits of ribosome proteins (*rpl2*, *rpl5*, and *rpl16*; in addition to chloroplast-derived *rpl2* and *rpl23*), ten small subunits of ribosome proteins (*rps1*, *rps2*, *rps3*, *rps4*, *rps7*, *rps11*, *rps12*, *rps13*, *rps14*, and *rps19*; in addition to a chloroplast-derived *rps7*).

### Repeats mediate the homologous recombination

During the assembly of Illumina short reads, we detected 20 repeat sequences in the initial graph (SR1-SR13, MR1-MR6, LR1) with multiple connections and doubled coverage depth, resulting in a complex mitochondrial genome structure (Fig. S4). Among them, except for LR1 and MR3, the rest of the repeat sequences have low recombination frequencies below 15% (Table 2). Furthermore, since there was no supporting evidence from ONT reads confirming the secondary configuration for SR8 and SR10, both of them were considered false positives or inactive in mediating recombination.

**Table 2:** Information on the 25 repeat sequences that potentially mediate homologous recombination in the mitochondrial genome

Repeat	Length (bp)	Direction	Conformations	Long reads support each conformation	Recombination rate (%)
SR1	392	+	m1: contig2-SR1-contig3	413	12.13
			m2: contig41-SR1-contig42	347	
			s1: contig2-SR1-contig42	12	
			s2: contig41-SR1-contig3	57	
SR2	278	+	m1: contig3-SR2-contig4	379	0.50
			m2: contig33-SR2-contig34	395	
			s1: contig3-SR2-contig34	2	
			s2: contig33-SR2-contig4	2	
SR3	238	+	m1: contig4-SR3-contig5	357	12.82
			m2: contig38-SR3-contig37	408	
			s1: contig4-SR3-contig37	23	
			s2: contig38-SR3-contig5	60	
SR4	210	+	m1: contig5-SR4-contig6	381	0.65
			m2: contig10-SR4-contig11	459	
			s1: contig5-SR4-contig11	3	
			s2: contig10-SR4-contig6	3	
SR5	385	-	m1: contig17-SR5-contig18	465	1.84
			m2: contig29-SR5-contig38	481	
			s1: contig17-SR5-contig38	1	
			s2: contig29-SR5-contig18	9	
SR6	191	+	m1: contig24-SR6-contig25	483	1.23
			m2: contig38-SR6-contig39	389	
			s1: contig24-SR6-contig39	0	
			s2: contig38-SR6-contig25	6	
SR7	377	+	m1: contig36-SR7-contig37	404	2.88
			m2: contig42-SR7-contig43	392	
			s1: contig36-SR7-contig43	2	
			s2: contig42-SR7-contig37	12	
SR8	181	+	m1: contig18-SR8-contig19	530	0.00
			m2: contig45-SR8-contig46	375	
			s1: contig18-SR8-contig46	0	
			s2: contig45-SR8-contig19	0	
SR9	448	-	m1: contig20-SR9-contig21	400	0.47
			m2: contig26-SR9-contig25	421	
			s1: contig20-SR9-contig25	1	
			s2: contig26-SR9-contig21	2	
SR10	128	-	m1: contig21-SR10-contig22	462	0.00
			m2: contig48-SR10-contig47	339	
			s1: contig21-SR10-contig47	0	
			s2: contig48-SR10-contig22	0	
SR11	136	-	m1: contig12-SR11-contig13	520	0.19
			m2: contig23-SR11-contig22	423	
			s1: contig12-SR11-contig22	0	
			s2: contig23-SR11-contig13	1	
SR12	337	+	m1: contig9-SR12-contig10	406	10.60
			m2: contig23-SR12-contig24	430	
			s1: contig9-SR12-contig24	2	
			s2: contig23-SR12-contig10	51	
SR13	475	+	m1: contig14-SR13-contig15	386	1.28
			m2: contig40-SR13-contig41	339	
			s1: contig14-SR13-contig41	4	
			s2: contig40-SR13-contig15	5	
MR1	513	+	m1: contig1-MR1-contig2	429	2.50
			m2: contig43-MR1-contig44	333	
			s1: contig1-MR1-contig44	9	
			s2: contig43-MR1-contig2	11	
MR2	794	-	m1: contig19-MR2-contig20	450	4.05
			m2: contig28-MR2-contig27	352	
			s1: contig19-MR2-contig27	19	
			s2: contig28-MR2-contig20	18	
MR3	553	-	m1: contig11-MR3-contig12	443	21.03
			m2: contig36-MR3-contig35	390	
			s1: contig11-MR3-contig35	118	
			s2: contig36-MR3-contig12	55	
MR4	574	-	m1: contig16-MR4-contig17	379	3.03
			m2: contig35-MR4-contig34	448	
			s1: contig16-MR4-contig34	14	
			s2: contig35-MR4-contig17	14	
MR5	858	+	m1: contig13-MR5-contig14	433	12.88
			m2: contig46-MR5-contig47	323	
			s1: contig13-MR5-contig47	26	
			s2: contig46-MR5-contig14	64	
MR6	911	-	m1: contig15-MR6-contig16	352	2.89
			m2: contig45-MR6-contig44	370	
			s1: contig15-MR6-contig44	8	
			s2: contig45-MR6-contig16	11	
LR1	1,769	-	m1: contig7-LR1-contig8	385	48.53
			m2: contig31-LR1-contig32	336	
			s1: contig7-LR1-contig32	219	
			s2: contig31-LR1-contig8	363	
CR1	5,692	+	m1: contig6-CR1-contig10	90	49.44
			m2: contig26-CR1-contig27	72	
			s1: contig6-CR1-contig27	88	
			s2: contig26-CR1-contig10	74	
CR2	19,407	-	m1: contig1-CR2-contig48	35	36.36
			m2: contig30-CR2-contig33	14	
			s1: contig1-CR2-contig33	20	
			s2: contig30-CR2-contig48	19	
CR3	12,002	Unknown	m1’: contig6-CR3-contig48	30	47.76
			m2’: contig26-CR3-contig33	35	
			s1: contig6-CR3-contig33	32	
			s2: contig26-CR3-contig48	25	
CR4	13,097	Unknown	m1’: contig1-CR4-contig27	46	47.73
			m2’: contig30-CR4-contig10	30	
			s1: contig1-CR4-contig10	34	
			s2: contig30-CR4-contig27	42	
CR5	7,561	-	m1: contig28-CR5-contig30	120	45.90
			m2: contig40-CR5-contig38	123	
			s1: contig28-CR5-contig38	113	
			s2: contig40-CR5-contig30	145	

Note: m1/m2: two paths representing the major conformation (i.e., “master ring” conformation); s1/s2: two paths representing the secondary conformation. SR (small repeat): < 500 bp; MR (middle repeat): > 500 bp & < 1000 bp; LR (large repeat): > 1000 bp; CR (complex repeat): repeat composed of multiple contigs. CR3 and CR4 were not present in the master ring conformation depicted in Fig. 1

**Fig. 2:**
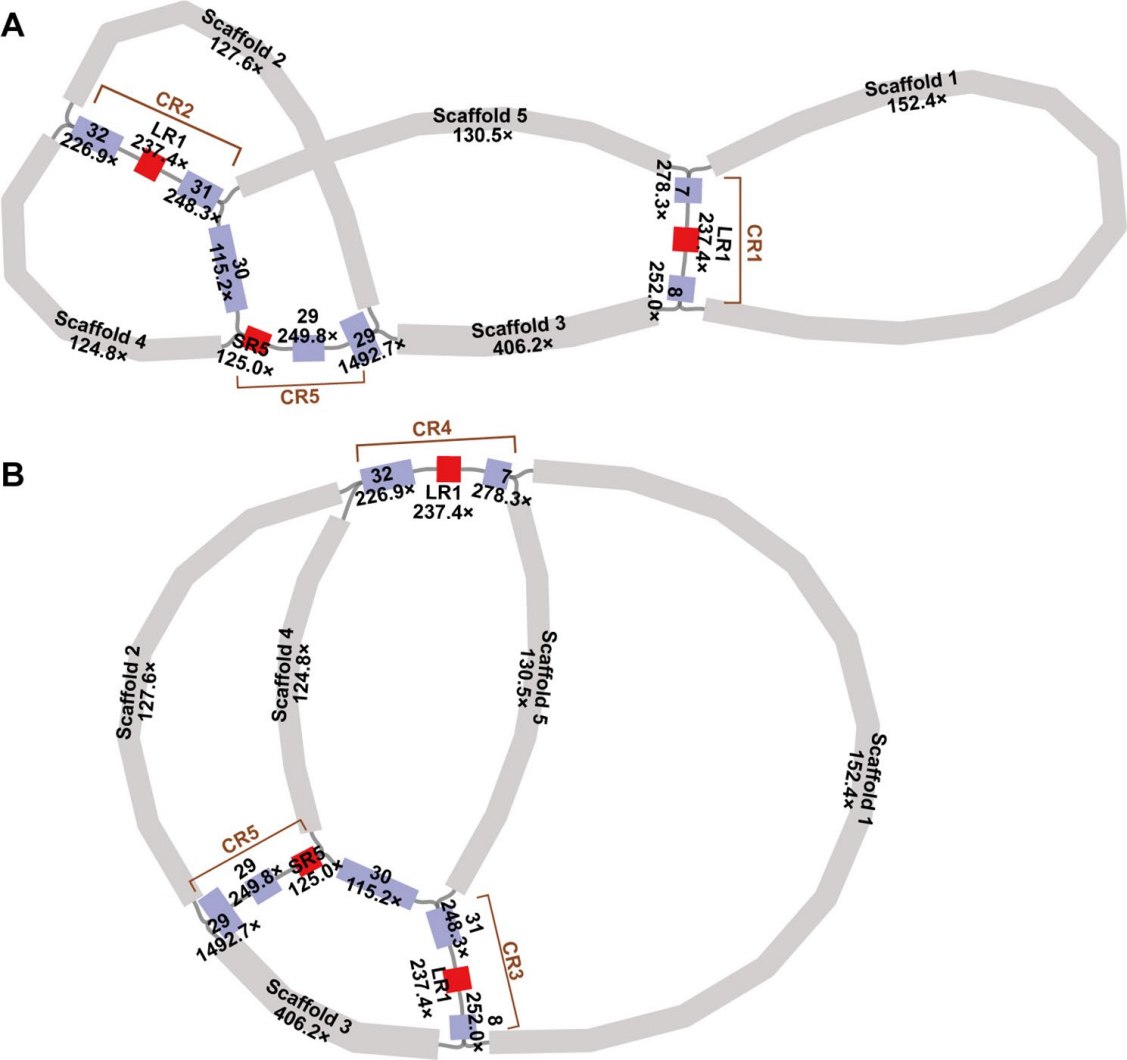
Demonstration of the two configurations mediated by LR1. (**A**) The main configuration shown in our master circle model. CR1: contig7-LR1-contig8; CR2: contig31-LR1-contig32; CR5: SR5-contig29. (**B**) The alternative configuration. CR3: contig8-LR1-contig31; CR4: contig7-LR1-contig32

The two configurations resulting from LR1 (major: contig7-LR1-contig8/contig31-LR1-contig32; secondary: contig7-LR1-contig32/contig31-LR1-contig8) occurred with almost equal frequency (385/363) (Fig. 2; Fig. S5; Table 2). In either configuration, LR1 can combine with other contigs to form component repeats (Fig. 2; Fig. S5). For example, in our master circle model, contig7-LR1-contig8 (CR1) and contig31-LR1-contig32 (CR2) (Fig. 2A), or contig8-LR1-contig31 (CR3) and contig7-LR1-contig32 (CR4) (Fig. 2B). The sequencing depth of the four aforementioned contigs is approximately twice the average depth (contig7: 278.3×; contig8: 252.0×; contig31: 248.3×; contig32: 226.9×). However, the depth of LR1 is four times the average (474.8×). Meanwhile, SR5-contig29 also forms a component repeat, namely CR5 (Fig. 2).

**Fig. 3:**
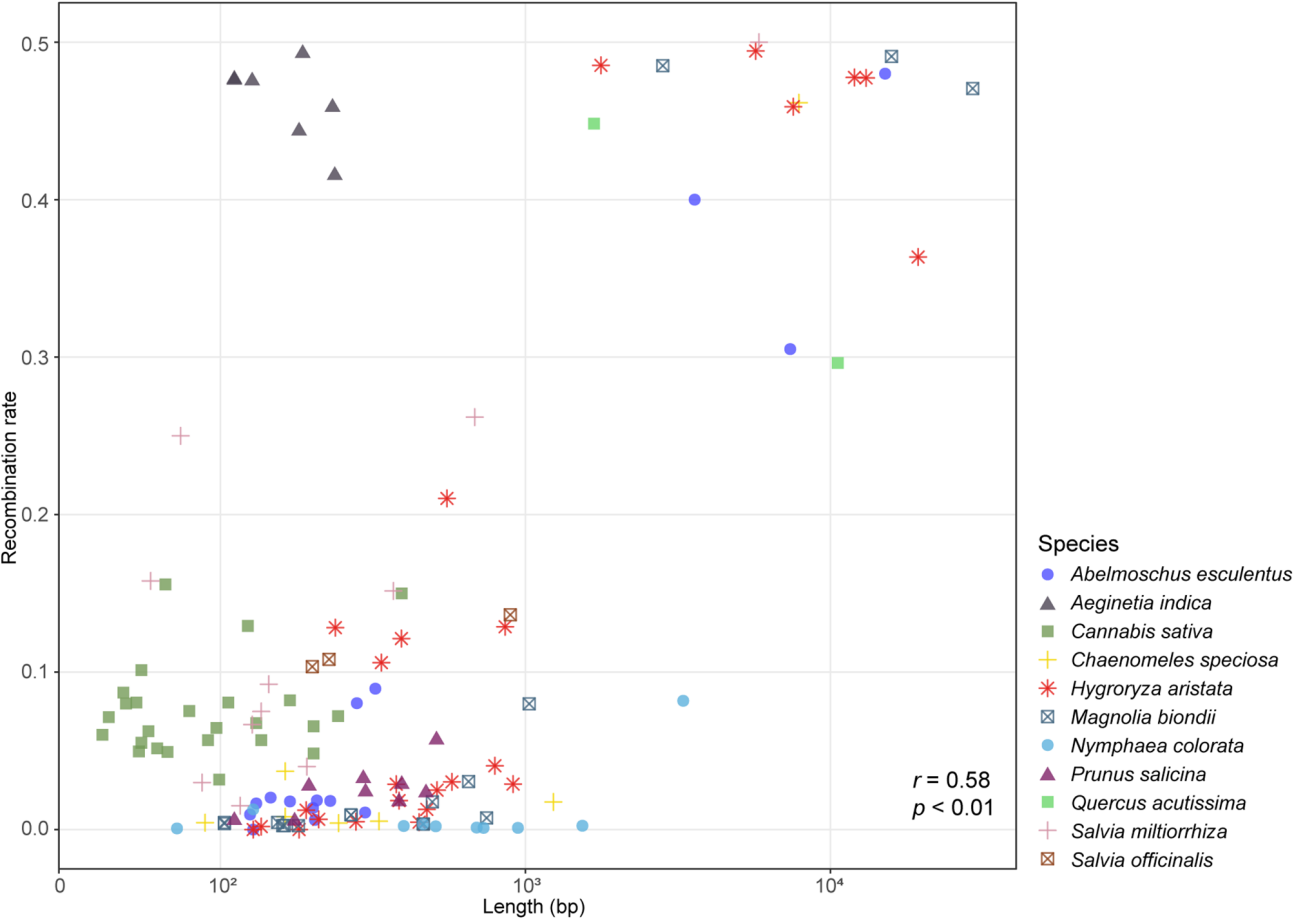
Dot plot depicting the length of repeat pairs and their homologous recombination rate. Species are differentiated by various colors and patterns

There is a clear positive correlation between the length of repeat sequences and their corresponding genome recombination rate (*r* = 0.58, *p* < 0.01; Fig. 3). Within floating bamboo, this correlation is even more pronounced (*r* = 0.74, *p* < 0.01). The six repeats exceeding 1000 bp (LR1, CR1-CR5) all exhibit high recombination rates (> 0.3; Table 2). Theoretically, these six repeats can lead to 64 different high-frequency configurations.

### Sequence exchange between genomes: MTCPs

**Fig. 4:**
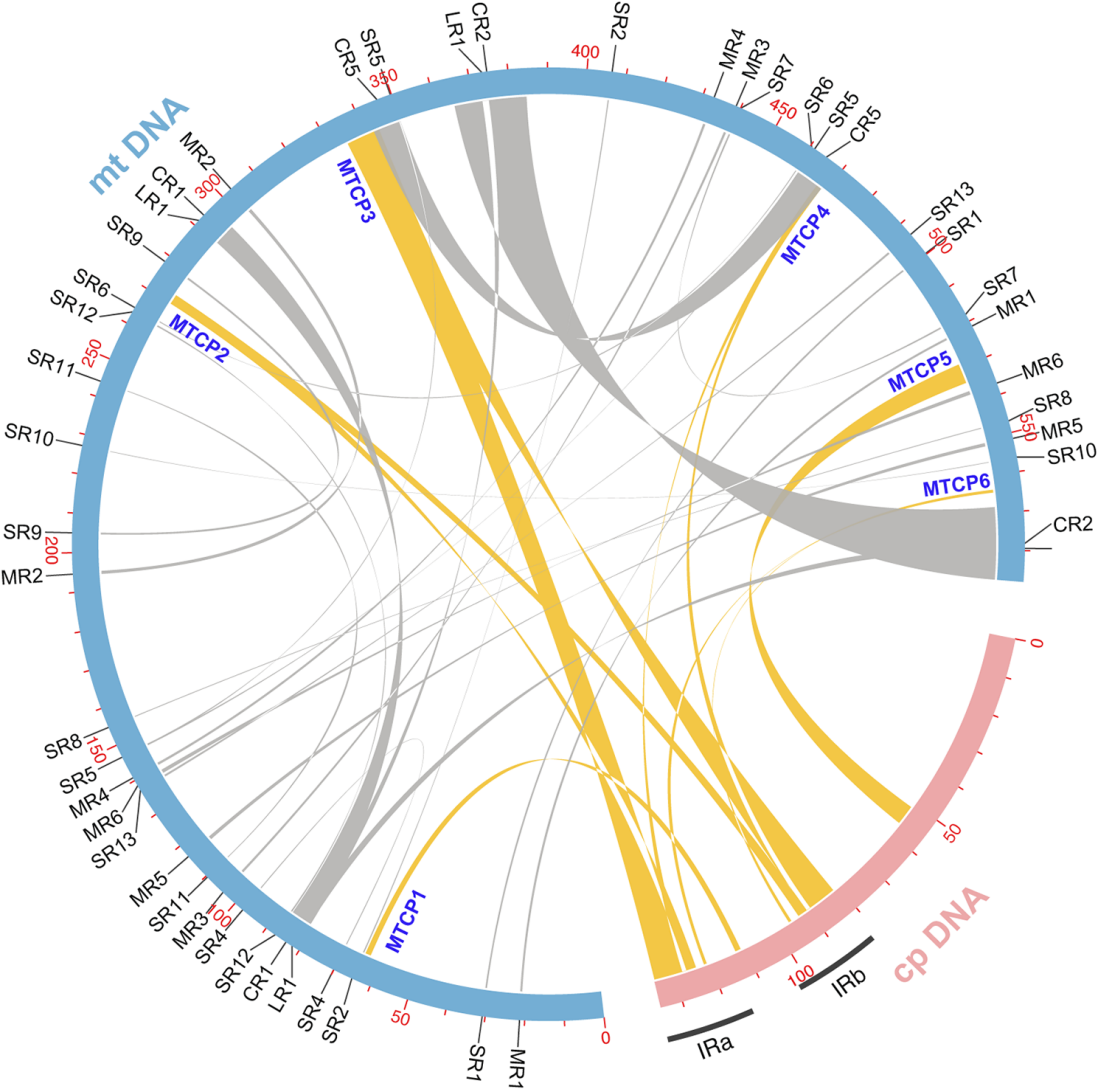
Demonstration of the homologous sequences between the two organelle genomes of *H. aristata*. The outer circle in blue and pink represents the mitogenome (mtDNA) and the plastome (cpDNA), respectively. Repeat sequences within the mitogenome and mitochondrial chloroplast DNAs (MTCPs) are indicated by gray and yellow arcs, respectively

During the process of evolution, mitochondrial genomes tend to incorporate segments from other intracellular genomes and integrate them into their genomes. Specific contigs of the mitochondrial genome display higher-than-average depth (contig25: 1723.0×; contig28: 1776.6×; contig29: 1492.7×; contig48: 1679.5×; Fig. S4), which are derived from the chloroplast genome. This suggests significant sequence transfer between these two organelles. By comparing the two organelles of *H. aristata*, we identified six mitochondrial chloroplast DNA sequences (MTCPs) (Fig. 4; Table S3), four of which originated from the inverted repeat (IR) regions of the chloroplast and corresponded to the high-depth contigs mentioned above. This explains why the depth of these contigs is greater than the sum of the average depths of the mt and cp genomes. The total length of these six MTCPs is 20,684 bp, accounting for 3.52% and 15.25% of the total length of the mt and cp genomes, respectively. Many intact genes inside these six long chloroplast fragments are transferred to the mitogenome. These transfers include nine protein-coding genes (*rpl2*, *rpl23*, *rps7*, *ndhI*, *ndhB*, *rbcL*, *ycf2*, *atpE*, and *atpB*), among which *atpE* and *atpB* have become pseudo-genes due to the introduction of stop codons through mutations. However, despite the presence of seven other complete cp PCGs in mtDNA, we have found no evidence of their expression in transcriptomic data.

### Repeat elements analysis

We identified 23 simple sequence repeats (SSRs) in the plastome and 145 in the mitogenome (Table S4). Dispersed repeats are more frequent than SSRs in both organelles, with 67 in the plastome and 681 in the mitogenome. Among them, there are 89 (13.07%) dispersed repeats larger than 1 kb in the mitogenome. Additionally, no reverse (R) or complement (C) repeats were detected. Relative to the high-frequency occurrence in the nuclear genome, tandem repeat elements have been found only 35 times in the mitochondrial genome of *H. aristata*, accounting for 0.51% of the total length of the mtDNA.

### Codon usage bias and RNA editing

The GC content of the PCGs in the mitogenome is as follows: GC1 (48.21%) > GC2 (42.84%) > GC3 (38.07%) (Table S5). Moreover, GC1 is notably higher than the average GC content of the entire genome (44.63%; Fig. 1). Although the two organelles share the same most frequent codon for each amino acid (Table S6), the effective number of codons (ENC) in the mitogenome remains significantly higher than that in the plastome (55.45 > 50.61; Table S5), indicating that the mitogenome exhibits a weaker codon preference. All PCGs are located near the expected curve in the GC3S-ENC plot, without significant outliers that deviate excessively from the expected values (Fig. S6B). In addition, there is a significant bias towards T/C bases at the third position of codons in the PCGs of both organelles (Fig. S6C).

When the RNA-sequencing data was mapped to the PCGs, it was observed that the average coverage of mt PCGs was only 3.72, while the corresponding value of cp PCGs was 59.90. This indicates a significantly lower expression level of mt PCGs compared to cp PCGs. Due to the low expression level of mt PCGs, only 2 mt genes with 3 editing sites were identified, which is much fewer than in chloroplast genes (14 genes with 61 editing sites; Table S7). Furthermore, the editing efficiency of these three editing sites in mt is all 1, suggesting the possibility of gene mutations between individuals cannot be ruled out.

### Phylogenetic and synteny analyses

**Fig. 5:**
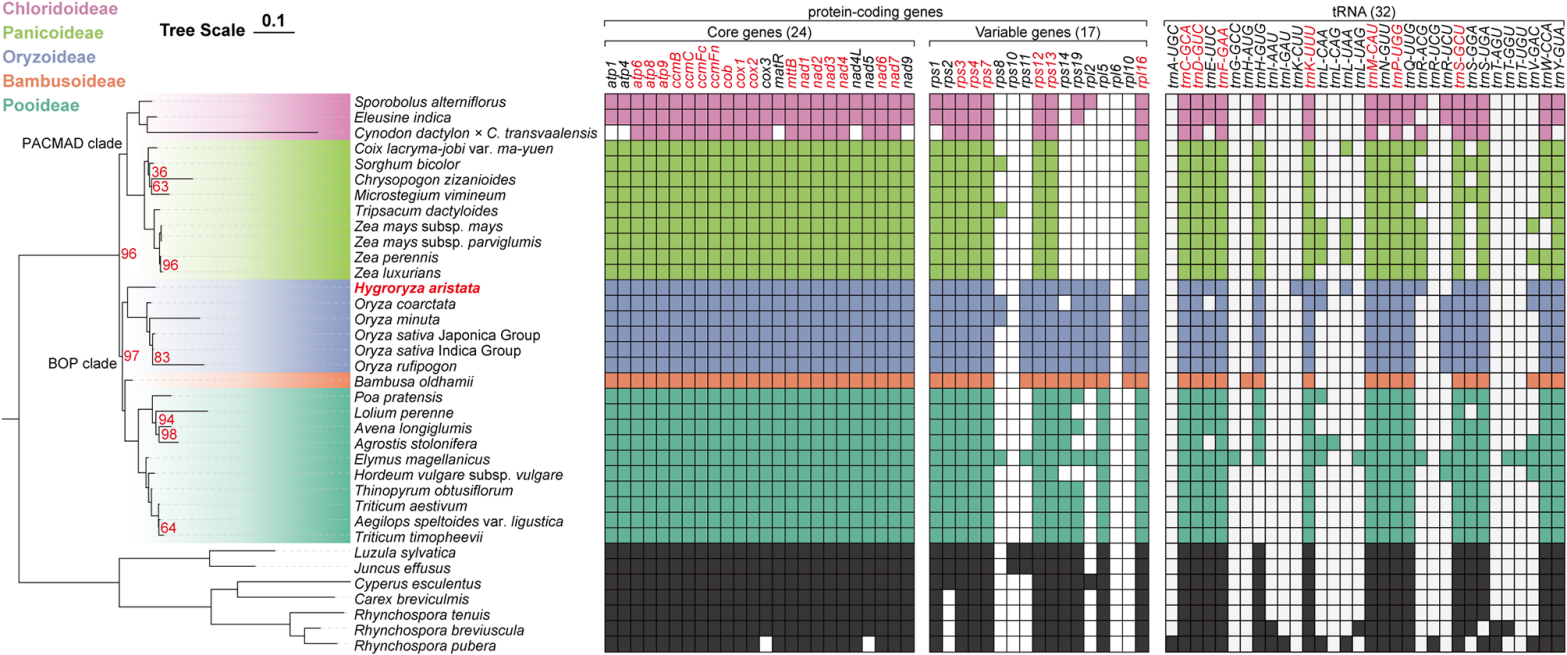
The phylogenetic relationships and gene map of Poales. (**A**) Maximum likelihood phylogeny of Poales inferred from IQ-TREE analysis of the concatenated 32 mitochondrial gene supermatrix. Branch lengths are in number of substitutions per site. All nodes have maximal support (IQ-TREE UFBoot = 100) unless noted with red-colored numbers. (**B**) Mitochondrial gene map of Poales where color-filled squares represent the presence of at least one complete copy. Species belonging to different subfamilies are color-coded for easy identification. Genes without absence in all species are highlighted in red. All tRNAs have scores exceeding 30.0 in tRNAscan-SE

The topology of the BOP clade, to which *H. aristata* belongs, was recovered as [Oryzoideae, (Bambusoideae, Pooideae)] with strong support (BS = 97) (Fig. 5). The majority of analyzed species contain a full set of 24 core unique mitochondrial genes. Furthermore, all species have retained the following genes: 3 rRNA genes, *rps3*, *rps4*, *rps7*, *rps12*, *rps13*, and *rpl16*. However, there is no presence of *sdh3* and *sdh4*. *Cynodon dactylon* × *C. transvaalensis* have lost the most genes, including five core genes (*atp1*, *atp4*, *matR*, *nad4L*, and *nad9*), and 10 variable genes, also exhibiting the fastest substitution rate in Poaceae. Regarding tRNA, only eight tRNA genes (*trnC-GCA, trnD-GUC, trnF-GAA, trnK-UUU, trnM-CAU, trnP-UGG,* and *trnS-GCU*) have not been lost in any of the species.

**Fig. 6:**
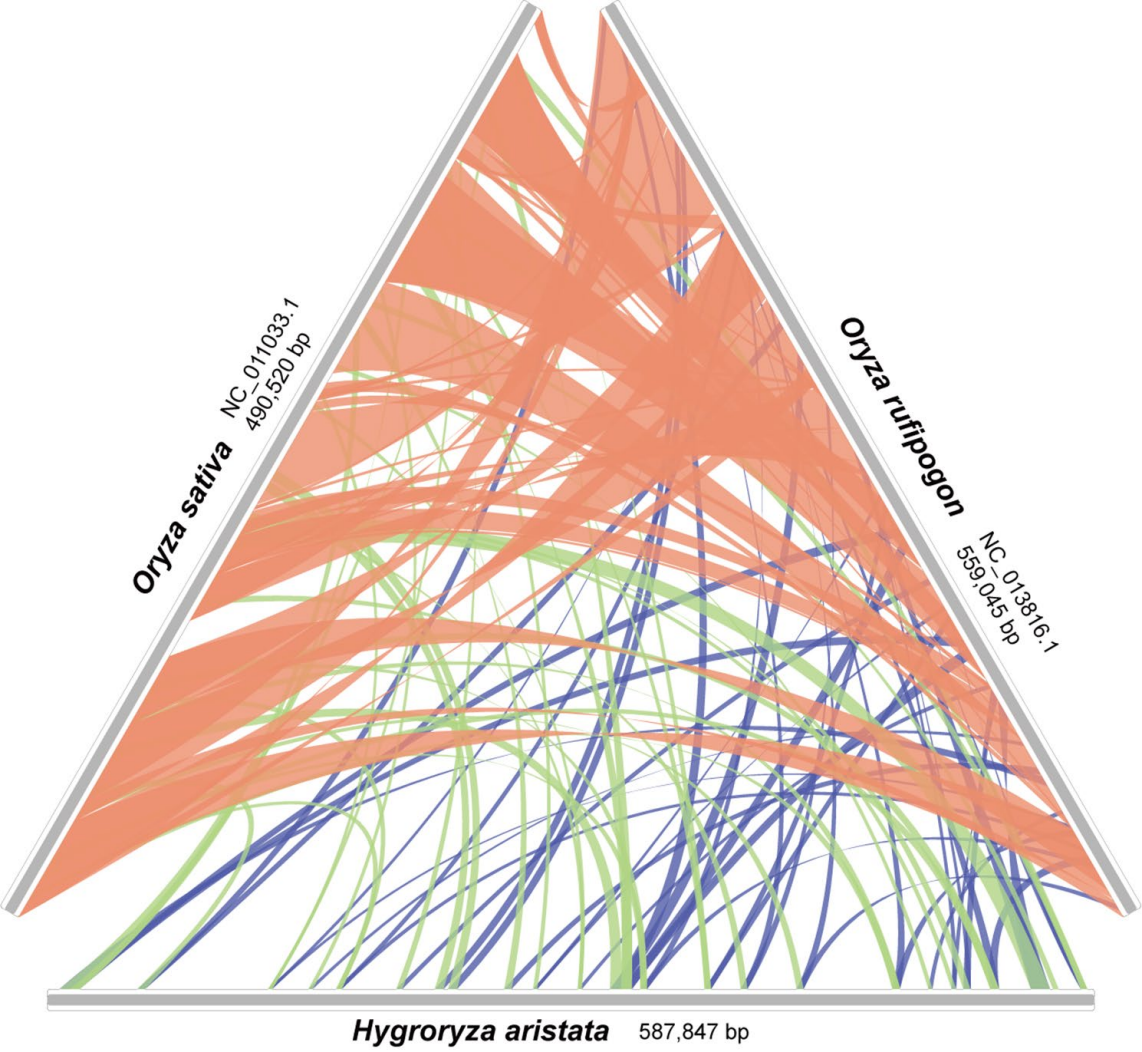
Collinearity between *Hygroryza aristata* and two *Oryza* species. Collinear segments less than 1 kb in length are not shown

Despite significant structural variations and rearrangements, there are abundant homologous sequences between the mitogenomes of *Oryza sativa* and *O. rufipogon*, with essentially no unique segments found in the latter (Fig. 6). Although *Hygroryza* and *Oryza* belong to the same subfamily, only 27.10% of sequences in the mitogenome of *H. aristata* show homology with *Oryza*, with the majority being in protein-coding regions.

## Discussion

In this study, the final assembly of the mitogenome was based solely on Illumina short reads, and Nanopore long reads were used to quantify the homologous recombination rate of each repeat pair (Table 2). As pointed out by Yang et al. (2022) [66], this approach can help avoid incorrect bases, especially in protein-coding regions, that may arise when constructing the scaffold using long reads [67]. While Unicycle [43] implements a similar strategy to the one used in this study for small genomes, it may overlook some repeat sequences with low recombination rates during the assembly process. Floating bamboo, as an endangered species, holds significant ecological and economic value. However, the lack of genetic information about this species hinders its conservation and research. Our presentation of its first complete mitochondrial genome enhances our understanding of this species and provides a critical case study for mitochondrial genomes in aquatic higher plants.

We detected 25 repeat pairs (13 SRs, 6 MRs, 1 LRs, and 5 CRs; Table 2) in the mitogenome of floating bamboo. Among these, except for SR8 and SR10, their activity in mediating homologous recombination has been proven. In addition to serving as sites for inter- and intra-molecular recombination, repeats also significantly inflate the estimated size of mtDNA. Except for CR3 and CR4, which do not appear in the master circle conformation, the length of the other repeat pairs reaches 81,633 bp, accounting for 13.88% of its total length. Many studies have confirmed that repeat pairs do not need to be identical to mediate HR [28, 34, 35]. However, we have not encountered this situation in floating bamboo. We have not found any precedent in former studies for the complex repeats we identified (CR1-CR5), undoubtedly adding to the complexity of plant mitogenomes. As one of the most important features in the mitochondria of land plants, homologous recombination mediated by repeats has been found in nearly all studied species. However, by losing two repeats, the mtDNA in *Brassica hirta* presents as a single circular chromosome with a smaller size compared to other *Brassica* species [26]. At the individual level, it seems preferable to keep the genome absolutely intact. However, a degree of instability is essential for species evolution, as genomic reshaping leads to genetic diversity. Disrupting the mechanisms would ultimately compromise the resilience and adaptability of species to changing environmental conditions [18].

Based on a review of previous studies, Marechal and Brisson (2010) [18] concluded that fairly large repeated sequences (> 1 kb) could frequently undergo intra- or intermolecular recombination, while homologous recombination also occurs sporadically between shorter repeats (> 100 bp, < 1 kb). As seen in Fig. 3, quantitative studies based on long sequencing reads generally support this conclusion. Most repeats with a recombination rate greater than 0.3 are longer than 1 kb. Except for *Aeginetia indica*, all seven repeats in this species range from 100 to 250 bp in length, yet their corresponding recombination ratios are greater than 0.4. In contrast, *Nymphaea colorata* exhibits a different pattern, as all ten repeats (4 SRs, 4 MRs, and 2 LRs) exhibit a low recombination rate (< 0.1). The differences between species are substantial, *Salvia miltiorrhiza*, compared to its congener *S. officinalis*, possesses more and longer repeat sequences and undergoes more frequent HR. Despite that, overall, the positive correlation between the length of repeat sequences and their corresponding genome recombination rate remains pronounced (*r* = 0.58, *p* < 0.01).

While horizontal gene transfer (HGT) between plant mitochondrial genomes and other intra- or extracellular genomes is frequently reported and contributes to the large size of plant mitogenomes [27, 68], there is currently no evidence confirming that these transferred exogenous genes can be expressed and functional within mitochondria. Fields et al. (2022) [69] reported a complete sequence insertion of 641 kb in the mtDNA of *Arabidopsis thaliana* from its nuclear genome. Li et al. (2022) [27] identified 28 MTCPs in okra, including only three complete chloroplast genes (*rps7*, *psaA*, and *psbJ*). Although we only identified six MTCP fragments in floating bamboo (Fig. 4), due to their long length, these fragments contain nine complete chloroplast protein-coding genes, with *atpE* and *atpB* undergoing pseudogenization (Table S3).

Our analysis confirmed that both organelle genomes of *H. aristata* exhibit the same pattern of codon usage bias. However, the PCGs in mtDNA show a more balanced usage of synonymous codons (Table S5 and S6). Furthermore, PCGs are distributed around the expected curve in the GC3S-ENC plot (Fig. S6B), consistent with the pattern summarized by Wang et al. (2011) [70]. They reported that during the evolution of the Streptophyta lineage, from Charophyta algae to the earliest land plant group (bryophytes) and finally to the highly derived and successful angiosperms (monocots and eudicots), the codon usages in their mitochondrial genomes have become less biased. They also suggested that the GC-accumulating strategy in the mitogenome of land plants may provide an advantage for DNA by reducing damage from strong ultraviolet radiation, as plants have gradually adapted to dry habitats over the course of their evolution.

It is a consensus that PCGs in mitogenomeare highly conserved in both quantity and genetics, contrasting with the complex and variable structure of its genome (Fig. 6). They are considered the most conservative among the three sets of genetic materials in angiosperms. The phylogenetic position of *H. aristata* based on them remains clear (Fig. 5) and is supported by previous studies based on nuclear and chloroplast datasets [9, 71].

Among a complete set of 41 PCGs in the mitogenome, 12 of them had been lost at least once in the 35 studied species (excluding *Cynodon dactylon* × *C. transvaalensis*). Gene loss is even more common in the chloroplasts of Poales. Wu et al. (2024) [72] observed that 30 out of 80 cp PCGs have been lost at least once in 93 Poales species, they speculate that the loss of the photosynthetic *ndh* gene may be an adaptation to the aquatic environment. Additionally, we observed that the aquatic lineage within Oryzoideae (*Oryza* and *H. aristata*) retains more genes related to ribosomal synthesis (*rpl* and *rps*). The reduction of cytoplasmic genomes is one of the prominent themes in eukaryotic genome evolution. However, it is rare for mitogenomes and plastomes to be lost entirely despite their ancient origins approximately 1–2 billion years ago. The parasitic plant *Viscum scurruloideum* possesses the smallest known mitochondrial genome among land plants, measuring only 66 kb [73]. With the reduction in size, there is a significant decrease in gene content. It has lost all nine mitochondrial *nad* genes, which are responsible for encoding the respiratory complex I (NADH dehydrogenase), an unprecedented occurrence in a multicellular organism. By comparing mitogenome content across the extant diversity of eukaryotes, researchers have inferred the evolutionary timing of mitochondrial gene loss events in different lineages [74–77]. In the most common ancestor of eukaryotes, the number of mitochondrial genes has reduced from over 1,000 genes estimated to have been present in the bacterial progenitor of mitochondria to 69 [12]. Subsequently, heterogeneous reduction occurred with variation both through time and across phylogenetic lineages, resulting in extant eukaryotes differing greatly in organelle gene content [74, 76]. In the sampled species we studied, *Cynodon dactylon* × *C. transvaalensis* has lost the highest number of genes, including *atp1*, *atp4*, *matR*, *nad4L*, *nad9*, *rps1, trnN-GUU, trnQ-UUG*, and *trnY-GUA*, which are unique to the Poales gene loss profile (Fig. 5). The mitogenome of the somatic hybrid *Solanum commersonii* × *S. tuberosum* also contains fewer genes than both parental species. Cho et al. (2022) [78] speculated that it may have originated from homologous recombination between the two parents.

## Conclusions

We have successfully cultured *Hygroryza aristata* using tissue culture, providing a new approach to conserving the germplasm resources of this endangered species. With the assistance of Nanopore long reads, we quantified the homologous recombination rate mediated by 25 repeat pairs (13 SRs, 6 MRs, 1 LR, and 5 CRs) and represented the mtDNA of floating bamboo as a master circle model. By incorporating former quantified studies from 10 other angiosperms, despite the variation among species, we confirmed a pronounced positive correlation between the length of repeats and their corresponding recombination rate. Among these species, floating bamboo exhibits the most complex conformations, with six repeat pairs exceeding 1000 bp in length showing an HR rate exceeding 0.3. Meanwhile, the complex repeat pairs discovered in this study are unprecedented and shed light on the evolution of mitochondrial genomes in aquatic higher plants, allowing deeper insights into the repeat-mediated recombination pattern in plant mitogenomes. The chloroplast insertions in *H. aristata* largely originate from the inverted repeat region and collectively contain nine complete protein-coding genes, among which two have undergone pseudogenization, while the remaining seven show no sign of expression in the mitogenome. Both organelle genomes exhibit similar codon usage bias, with the PCGs in mtDNA showing a more balanced usage of synonymous codons. The gene map of the Poales mitogenome indicates the complete loss of *rpl6*, *sdh3*, and *sdh4* genes, with Oryzoideae retaining a greater number of variable genes compared to Pooideae.

### Electronic supplementary material

Below is the link to the electronic supplementary material.

Supplementary Material 1

Supplementary Material 2

## Data Availability

The assembled organelle genome sequences have been deposited in NCBI with accession number: PP534167 (mtDNA); PP534166 (cpDNA). The raw sequence data have been deposited in the Short Read Achieve (SRA) database of NCBI (PRJNA1090843).

## Abbreviations

HR: Homologous recombination
PCG: Protein-coding gene
cpDNA: Chloroplast DNA
mtDNA: Mitochondrial DNA
MTCP: Mitochondrial chloroplast DNA sequence
ENC: Effective number of codons
SR: Small repeat (< 500 bp)
MR: Middle repeat (> 500 bp, < 1 kb)
LR: Large repeat (> 1 kb)
CR: Complex repeat
