# Supplementary material for: Highly active repeat-mediated recombination in the mitogenome of the aquatic grass Hygroryza aristata
Source: BMC Plant Biol. 2024 Jul 8;24:644. doi: 10.1186/s12870-024-05331-x (PMC11229283; doi:10.1186/s12870-024-05331-x)
Supplement: Supplementary file 1 — Supplementary Material 1 [file 12870_2024_5331_MOESM1_ESM.docx]

**Supplementary Information**

**Highly active repeat-mediated recombination in the mitogenome of the aquatic grass *Hygroryza aristata***

Huijun Wang^1,2^, Zhigang Wu^1,2*^, Tao Li^1,2*^, Jindong Zhao^1,3^

This file contains:

- **6 supplementary figures (S1–S6)**

**
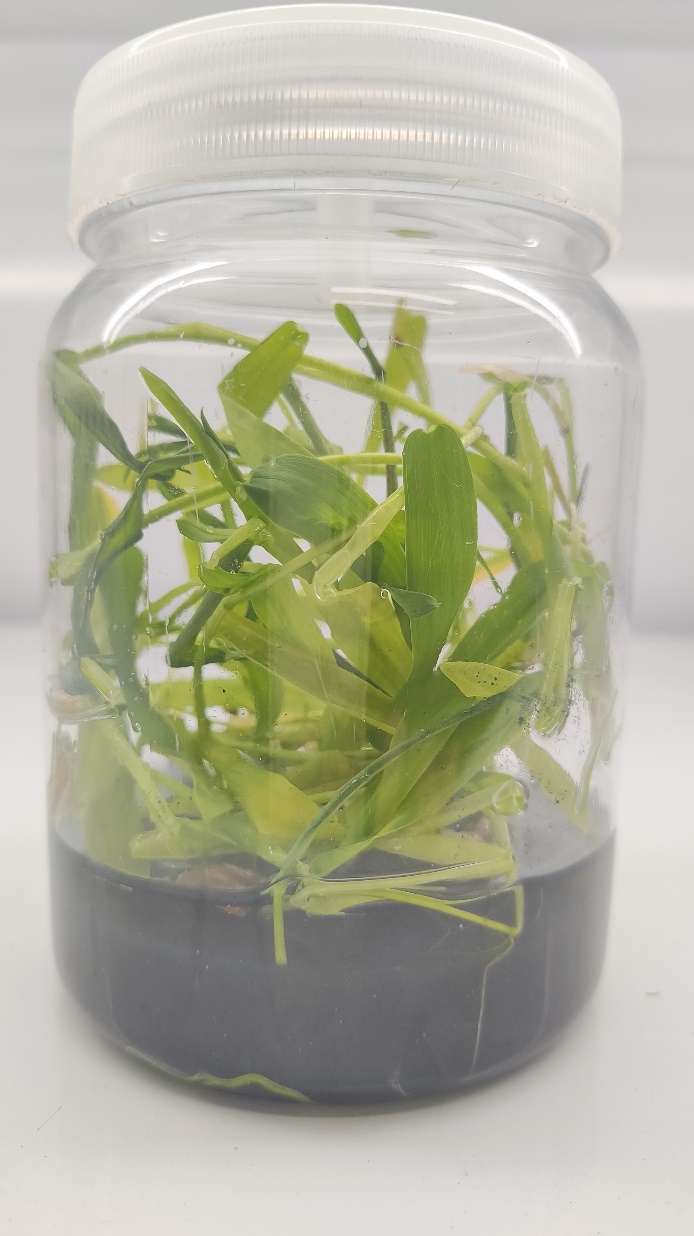
**

**Fig. S1** *H. aristata* cultivated on MS medium*.*

**
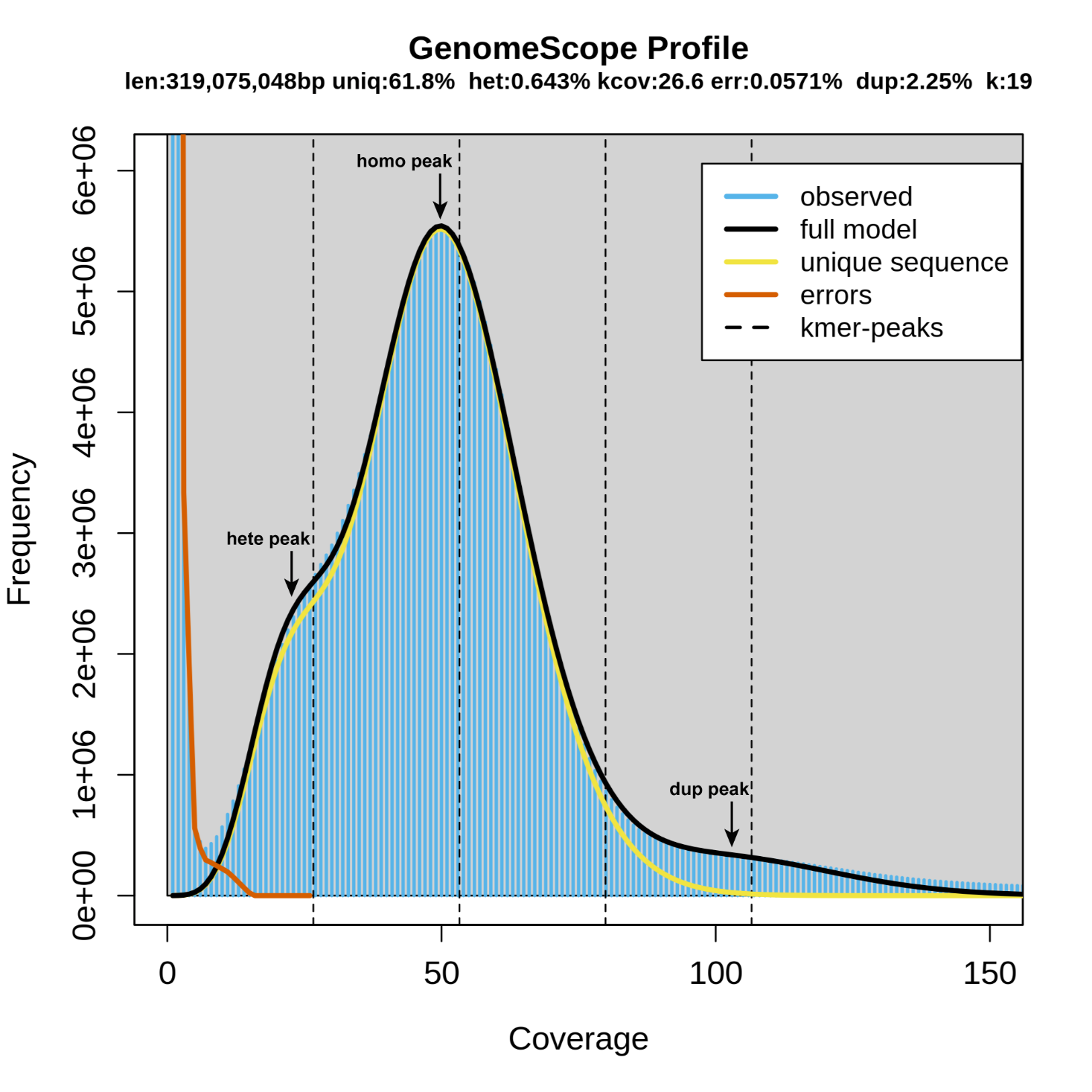
**

**Fig. S2** *K*-mer distribution of Illumina sequencing reads. The genome size was estimated as 319 Mb. Hete peak, the heterozygous peak refers to the peak value of heterozygous *k*-mer profile located at the heterozygous region of genome; homo peak, the homozygous peak refers to the peak value of homozygous *k*-mer profile located at the homozygous region of genome; dup peak, the duplicated peak shows the peak value of duplicated *k*-mer profile located at the duplicated region of genome.


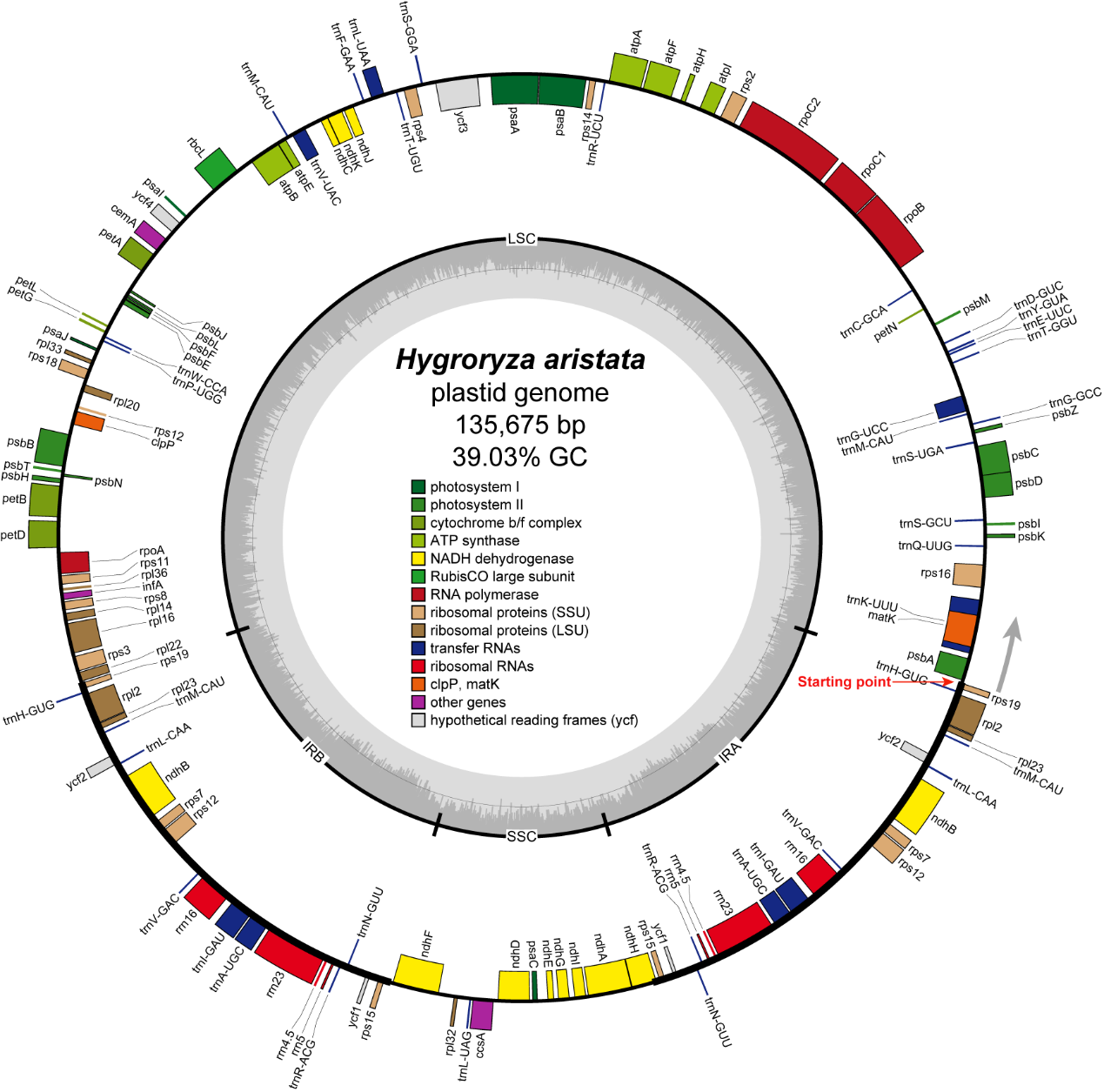


**Fig. S3** A representative chloroplast genome map of *H. aristata*.

**
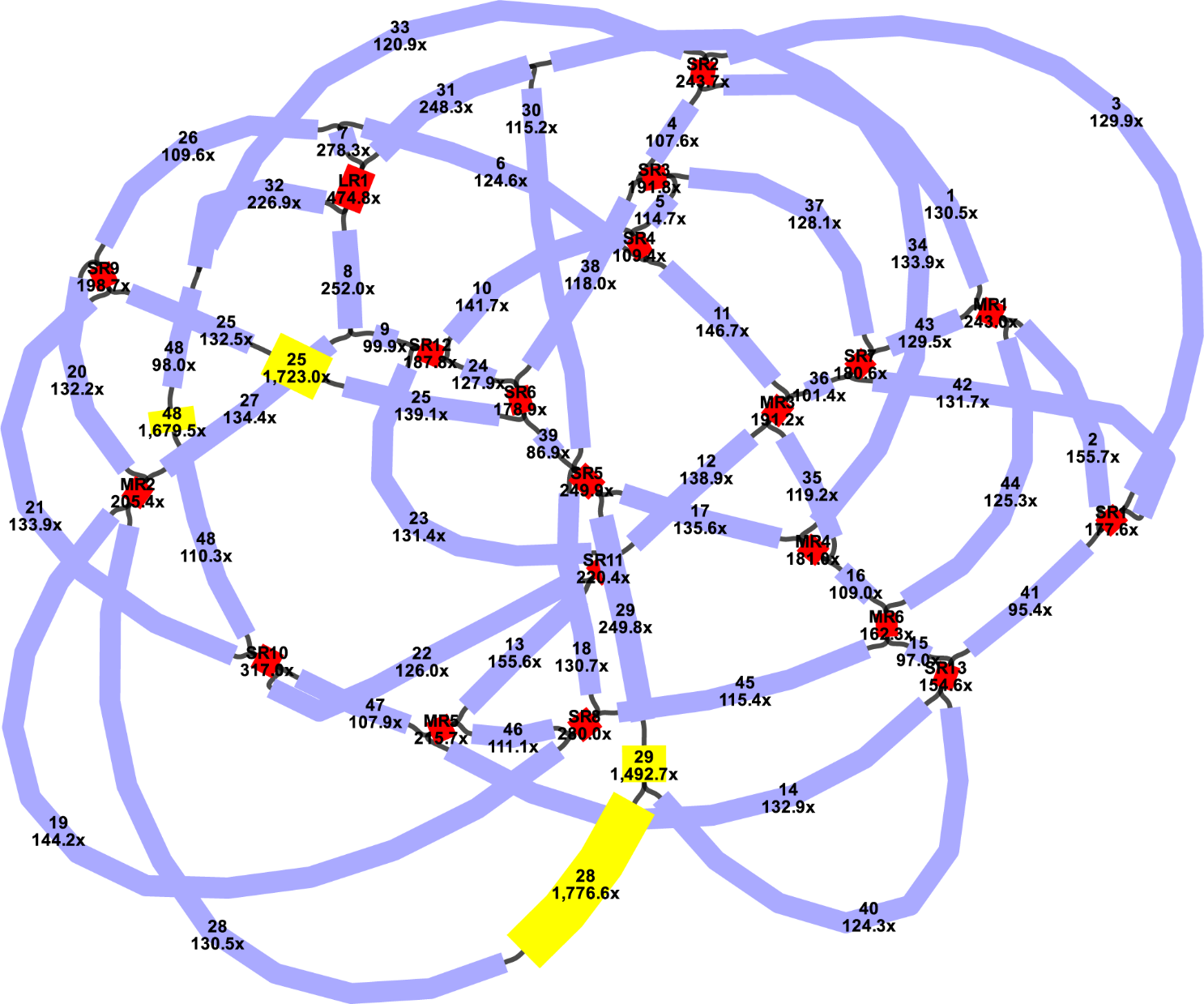
**

**Fig. S4** The primary structure of *H. aristata* mitogenome obtained from Illumina short reads. The colorful bars represent different segments of the mitogenome. Ordinary contigs are depicted in purple, chloroplast-derived contigs with higher-than-average coverage depth are shown in yellow, and repeat sequences that potentially mediate homologous recombination (SR1-SR13, MR1-MR6, LR1) are shown in red. The length and width of the bars correspond to the lengths and coverage depth of these contigs, respectively. Additionally, the black lines represent the potential linkage patterns between the sequences.

**
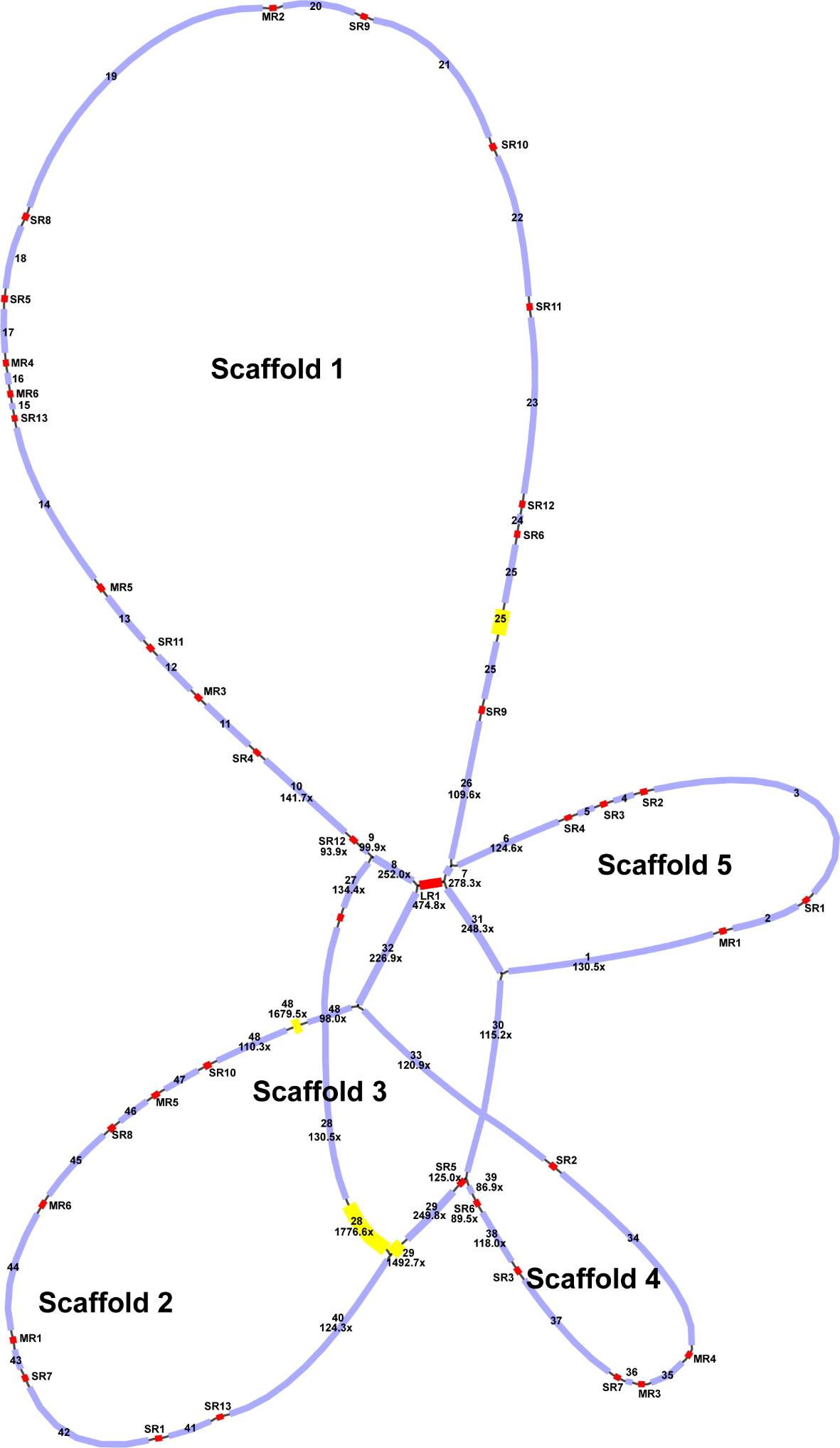
**

**Fig. S5** The mitochondrial structure after resolving 13 SRs and 6 MRs using Nanopore long reads, as detailed in Table 2.


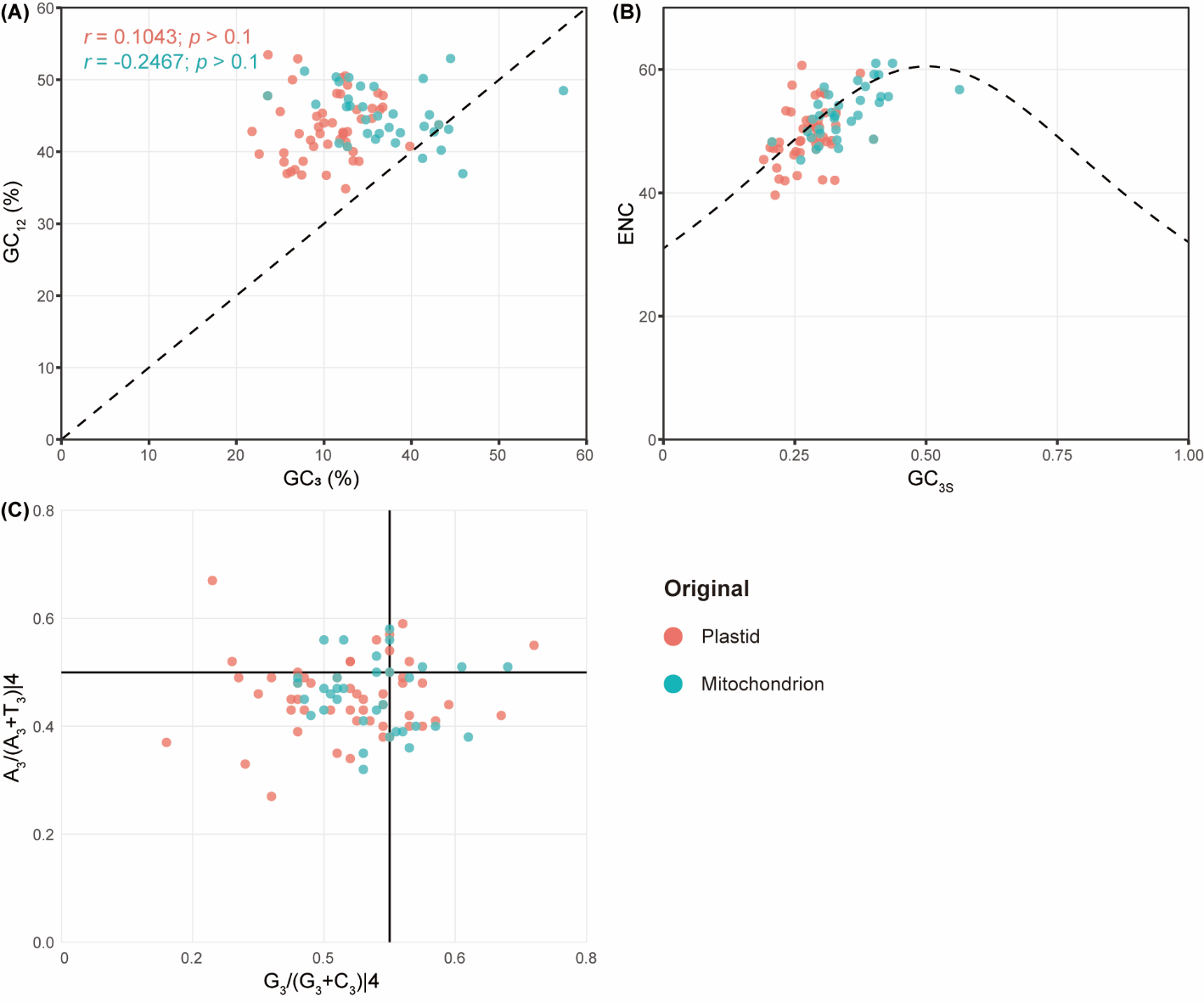


**Fig. S6** The evolutionary forces analyses of organelle genes. (A) Neutrality plot (GC_3_ vs GC_12_). (B) ENC plot (GC_3S_ vs ENC). (C) PR2-bias plot (G_3_/(G_3_ +C_3_) vs A_3_/(A_3_ + T_3_)).
