# Supplementary material for: Highly active repeat-mediated recombination in the mitogenome of the aquatic grass Hygroryza aristata
Source: BMC Plant Biol. 2024 Jul 8;24:644. doi: 10.1186/s12870-024-05331-x (PMC11229283; doi:10.1186/s12870-024-05331-x)
Supplement: Supplementary file 2 — Supplementary Material 2 [file 12870_2024_5331_MOESM2_ESM.docx]

**Supplementary Information**

**Highly active repeat-mediated recombination in the mitogenome of the aquatic grass *Hygroryza aristata***

Huijun Wang^1,2^, Zhigang Wu^1,2*^, Tao Li^1,2*^, Jindong Zhao^1,3^

This file contains:

- **7 supplementary tables (S1–S7)**

**Table S1 The information of the complete mitochondrial genome of Poales published on NCBI.**

| Abbreivation | Species | Family | Clade | Subfamily | Len (bp) | Topology |
| --- | --- | --- | --- | --- | --- | --- |
| NC_069587.1 | *Luzula sylvatica* | Juncaceae | Juncaceae | Juncaceae | 633,356 | circular |
| NC_069588.1 | *Juncus effusus* | Juncaceae | Juncaceae | Juncaceae | 519,026 | circular |
| NC_058697.1 | *Cyperus esculentus* | Cyperaceae | Cyperaceae | Cyperaceae | 1,002,696 | circular |
| NC_068215.1 | *Rhynchospora breviuscula* | Cyperaceae | Cyperaceae | Cyperaceae | 2,222,920 | circular |
| NC_068216.1 | *Rhynchospora pubera* | Cyperaceae | Cyperaceae | Cyperaceae | 2,064,773 | circular |
| NC_068217.1 | *Rhynchospora tenuis* | Cyperaceae | Cyperaceae | Cyperaceae | 1,678,054 | circular |
| NC_068626.1 | *Carex breviculmis* | Cyperaceae | Cyperaceae | Cyperaceae | 1,414,795 | circular |
| EU365401.1 | *Bambusa oldhamii* | Poaceae | BOP | Bambusoideae | 509,941 | circular |
| MG429050.1 | *Oryza coarctata* | Poaceae | BOP | Oryzoideae | 491,065 | circular |
| NC_007886.1 | *Oryza sativa Indica Group* | Poaceae | BOP | Oryzoideae | 491,515 | circular |
| NC_011033.1 | *Oryza sativa Japonica Group* | Poaceae | BOP | Oryzoideae | 490,520 | linear |
| NC_013816.1 | *Oryza rufipogon* | Poaceae | BOP | Oryzoideae | 559,045 | circular |
| NC_029816.1 | *Oryza minuta* | Poaceae | BOP | Oryzoideae | 515,022 | circular |
| JX999996.1 | *Lolium perenne* | Poaceae | BOP | Pooideae | 678,580 | circular |
| MN127966.1 | *Hordeum vulgare subsp. vulgare* | Poaceae | BOP | Pooideae | 525,599 | circular |
| NC_022666.1 | *Aegilops speltoides var. ligustica* | Poaceae | BOP | Pooideae | 476,091 | circular |
| NC_022714.1 | *Triticum timopheevii* | Poaceae | BOP | Pooideae | 443,419 | circular |
| NC_036024.1 | *Triticum aestivum* | Poaceae | BOP | Pooideae | 452,526 | circular |
| NC_072709.1 | *Elymus magellanicus* | Poaceae | BOP | Pooideae | 583,450 | circular |
| NC_072961.1 | *Avena longiglumis* | Poaceae | BOP | Pooideae | 548,445 | circular |
| NC_077587.1 | *Poa pratensis* | Poaceae | BOP | Pooideae | 447,463 | circular |
| NC_082434.1 | *Agrostis stolonifera* | Poaceae | BOP | Pooideae | 560,800 | circular |
| OK120846.1 | *Thinopyrum obtusiflorum* | Poaceae | BOP | Pooideae | 390,725 | circular |
| MK175054.1 | *Cynodon dactylon × C. transvaalensis* | Poaceae | PACMAD | Chloridoideae | 366,612 | circular |
| MT471321.1 | *Sporobolus alterniflorus* | Poaceae | PACMAD | Chloridoideae | 566,328 | circular |
| NC_040989.1 | *Eleusine indica* | Poaceae | PACMAD | Chloridoideae | 520,691 | circular |
| MT471097.1 | *Coix lacryma-jobi var. ma-yuen* | Poaceae | PACMAD | Panicoideae | 660,150 | circular |
| NC_007982.1 | *Zea mays subsp. mays* | Poaceae | PACMAD | Panicoideae | 569,630 | circular |
| NC_008331.1 | *Zea perennis* | Poaceae | PACMAD | Panicoideae | 570,354 | circular |
| NC_008332.1 | *Zea mays subsp. parviglumis* | Poaceae | PACMAD | Panicoideae | 680,603 | circular |
| NC_008333.1 | *Zea luxurians* | Poaceae | PACMAD | Panicoideae | 539,368 | circular |
| NC_008360.1 | *Sorghum bicolor* | Poaceae | PACMAD | Panicoideae | 468,628 | circular |
| NC_008362.1 | *Tripsacum dactyloides* | Poaceae | PACMAD | Panicoideae | 704,100 | circular |
| NC_056367.1 | *Chrysopogon zizanioides* | Poaceae | PACMAD | Panicoideae | 551,622 | circular |
| NC_072666.1 | *Microstegium vimineum* | Poaceae | PACMAD | Panicoideae | 478,010 | circular |

**Table S2 Sequencing information of *H. aristata*.**

|  | **Total reads** | **Total base (Gb)** | **Average length (bp)** | **GC content (%)** | **Accession Numbers** |
| --- | --- | --- | --- | --- | --- |
| **Illumina** | 174,437,368 | 23.60 | 135 | 44.55 | SRX24021652 |
| **Nanopore** | 1,441,833 | 22.54 | 15,631 | 43.52 | SRX24021653 |

**Table S3 Homologous sequences identified in the mitochondrial and plastid genomes (MTPTs) of *H. aristata*.**

| **Name** | **Aligned length (bp)** | **Identity (%)** | **Mitochondrial** | |  | **Chloroplast** | | **Location** | **Contained gene** |
| --- | --- | --- | --- | --- | --- | --- | --- | --- | --- |
|  |  |  | **Start** | **End** |  | **Start** | **End** |  |  |
| **MTPT1** | 1,456 | 89.72 | 62,922 | 64,377 |  | 111,074 | 112,547 | SSC | *ndhI*, *ndhA* (intron2) |
| **MTPT2** | 2,645 | 99.74 | 270,054 | 272,698 |  | 93,211 | 90,571 | IR-b | *trnV-GAC*, *rrn16* (partial) |
|  | 2,645 | 99.74 | 270,054 | 272,698 |  | 123,993 | 126,633 | IR-a |  |
| **MTPT3** | 9,139 | 99.95 | 334,629 | 343,767 |  | 81,665 | 90,803 | IR-b | *rps19* (partial), *trnH-GUG, rpl2*, *rpl23*, *trnM-CAU,* *ycf2*, *trnL-CAA*, *ndhB*, *rps7*, *rps12* (intron2, intron3) |
|  | 9,139 | 99.95 | 334,629 | 343,767 |  | 135,539 | 126,401 | IR-a |  |
| **MTPT4** | 1,423 | 99.79 | 467,234 | 468,656 |  | 90,803 | 89,381 | IR-b | *rps12* (intron2, intron3) |
|  | 1,423 | 99.79 | 467,234 | 468,656 |  | 126,401 | 127,823 | IR-a |  |
| **MTPT5** | 5,279 | 91.79 | 528,966 | 534,244 |  | 51,127 | 56,426 | LSC | *trnM-CAU*, *atpE**, *atpB**, *rbcL* |
| **MTPT6** | 742 | 99.06 | 563,785 | 564,526 |  | 95,539 | 96,280 | IR-b | *trnA-UGC* (partial)*, rrn23* (partial) |
|  | 742 | 99.06 | 563,785 | 564,526 |  | 121,665 | 120,924 | IR-a |  |

Note: * indicates the presence of stop codon in the middle, resulting in incomplete genes in mitochondrial genome.

**Table S4 Different types of repeat elements identified in the organelles.**

|  | **Simple sequence repeat** | | | | | |  | **Dispersed repeat** | | | |  | **Tandem repeat element** | | |
| --- | --- | --- | --- | --- | --- | --- | --- | --- | --- | --- | --- | --- | --- | --- | --- |
|  | **mono-** | **di-** | **tri-** | **tetra-** | **penta-** | **hexa-** |  | **F** | **R** | **P** | **C** |  | **Number** | **Length (bp)** | **Proportion (%)** |
| **CP** | 8 | 4 | 2 | 9 | 0 | 0 |  | 43 | 0 | 24 | 0 |  | 31 | 1758 | 1.2957 |
| **MT** | 33 | 33 | 23 | 51 | 4 | 1 |  | 361 | 0 | 320 | 0 |  | 35 | 2984 | 0.5076 |

**Table S5** **Parameter statistics of codon usage analyses for the organelle genomes in *H. aristata*.**

|  | **GCall** | **GC1** | **GC2** | **GC3** | **GC3s** | **L_aa** | **L_sym** | **CAI** | **CBI** | **Fop** | **ENC** | **Gravy** | **Aromo** |
| --- | --- | --- | --- | --- | --- | --- | --- | --- | --- | --- | --- | --- | --- |
| **CP** | 39.42 | 47.75 | 39.38 | 31.11 | 28.13 | 16985 | 16227 | 0.167 | -0.089 | 0.358 | 50.607 | -0.005 | 0.113 |
| **MT** | 43.04 | 48.21 | 42.84 | 38.07 | 35.48 | 11229 | 10742 | 0.17 | -0.063 | 0.372 | 55.448 | 0.045 | 0.110 |

Note: Gcall: the overall GC content. GC1/2/3: the GC content at the first, second, and third base position of the codons. GC3s: the GC content at the third base position of synonymous codons (excluding Met, Trp, and three stop codons). L_aa: total number of amino acid (excluding stop codons). L_sym: number of synonymous codons. CAI: Codon Adaptation Index. CBI: Codon bias index. Fop: Frequency of OPtimal codons. ENC: Effective Number of Codons. Gravy: grand average of hydropathicity.

**Table S6 RSCU values for the organelle genomes in *H. aristata*. The most frequent codon for each amino acid is highlighted in blue.**

| **AA** | **Codon** | **CP** | | **MT** | |
| --- | --- | --- | --- | --- | --- |
|  |  | ***No.*** | **RSCU** | ***No.*** | **RSCU** |
| **Ala** | GCA | 306 | 1.15 | 192 | 1.04 |
|  | GCC | 159 | 0.60 | 163 | 0.88 |
|  | GCG | 137 | 0.51 | 104 | 0.56 |
|  | GCU | 464 | 1.74 | 280 | 1.52 |
| **Arg** | AGA | 285 | 1.66 | 192 | 1.49 |
|  | AGG | 110 | 0.64 | 104 | 0.81 |
|  | CGA | 223 | 1.30 | 148 | 1.15 |
|  | CGC | 91 | 0.53 | 74 | 0.57 |
|  | CGG | 88 | 0.51 | 96 | 0.74 |
|  | CGU | 234 | 1.36 | 160 | 1.24 |
| **Asn** | AAC | 178 | 0.53 | 119 | 0.67 |
|  | AAU | 488 | 1.47 | 238 | 1.33 |
| **Asp** | GAC | 145 | 0.45 | 113 | 0.61 |
|  | GAU | 505 | 1.55 | 260 | 1.39 |
| **Cys** | UGC | 50 | 0.55 | 69 | 0.81 |
|  | UGU | 133 | 1.45 | 102 | 1.19 |
| **Gln** | CAA | 458 | 1.52 | 261 | 1.54 |
|  | CAG | 143 | 0.48 | 79 | 0.46 |
| **Glu** | GAA | 677 | 1.48 | 338 | 1.40 |
|  | GAG | 236 | 0.52 | 144 | 0.60 |
| **Gly** | GGA | 486 | 1.54 | 284 | 1.42 |
|  | GGC | 129 | 0.41 | 109 | 0.55 |
|  | GGG | 257 | 0.81 | 156 | 0.78 |
|  | GGU | 390 | 1.24 | 250 | 1.25 |
| **His** | CAC | 97 | 0.50 | 60 | 0.44 |
|  | CAU | 291 | 1.50 | 212 | 1.56 |
| **Ile** | AUA | 424 | 0.92 | 237 | 0.84 |
|  | AUC | 267 | 0.58 | 248 | 0.88 |
|  | AUU | 698 | 1.51 | 357 | 1.27 |
| **Leu** | CUA | 265 | 0.85 | 170 | 0.86 |
|  | CUC | 129 | 0.42 | 128 | 0.65 |
|  | CUG | 96 | 0.31 | 111 | 0.56 |
|  | CUU | 407 | 1.31 | 266 | 1.35 |
|  | UUA | 625 | 2.01 | 298 | 1.51 |
|  | UUG | 343 | 1.10 | 211 | 1.07 |
| **Lys** | AAA | 606 | 1.47 | 317 | 1.20 |
|  | AAG | 217 | 0.53 | 213 | 0.80 |
| **Met** | AUG | 387 | 1.00 | 283 | 1.00 |
| **Phe** | UUC | 332 | 0.69 | 305 | 0.86 |
|  | UUU | 625 | 1.31 | 402 | 1.14 |
| **Pro** | CCA | 195 | 1.09 | 170 | 1.17 |
|  | CCC | 177 | 0.99 | 125 | 0.86 |
|  | CCG | 82 | 0.46 | 83 | 0.57 |
|  | CCU | 261 | 1.46 | 202 | 1.39 |
| **Ser** | AGC | 80 | 0.40 | 108 | 0.67 |
|  | AGU | 253 | 1.26 | 165 | 1.02 |
|  | UCA | 210 | 1.04 | 181 | 1.12 |
|  | UCC | 236 | 1.17 | 172 | 1.06 |
|  | UCG | 108 | 0.54 | 125 | 0.77 |
|  | UCU | 320 | 1.59 | 223 | 1.37 |
| **Ter** | UAA | 27 | 1.59 | 13 | 1.15 |
|  | UAG | 12 | 0.71 | 11 | 0.97 |
|  | UGA | 12 | 0.71 | 10 | 0.88 |
| **Thr** | ACA | 241 | 1.08 | 143 | 0.96 |
|  | ACC | 172 | 0.77 | 146 | 0.98 |
|  | ACG | 109 | 0.49 | 85 | 0.57 |
|  | ACU | 371 | 1.66 | 219 | 1.48 |
| **Trp** | UGG | 320 | 1.00 | 170 | 1.00 |
| **Tyr** | UAC | 143 | 0.45 | 92 | 0.53 |
|  | UAU | 492 | 1.55 | 257 | 1.47 |
| **Val** | GUA | 359 | 1.46 | 195 | 1.15 |
|  | GUC | 122 | 0.50 | 115 | 0.68 |
|  | GUG | 132 | 0.54 | 154 | 0.91 |
|  | GUU | 370 | 1.51 | 212 | 1.25 |

**Table S7 RNA editing sites identified in the organelles of *H. aristata*.**

| **Source** | **Gene** | **Position (bp)** | **Coverage (×)** | **Ref** | **Sub** | **Editing efficiency** | **Codon change** | **AA change** | **Type** |
| --- | --- | --- | --- | --- | --- | --- | --- | --- | --- |
| **CP** | *atpA* | 1148 | 49 | C | U | 1.00 | TCA→TUA | S→L | non- |
|  | *atpI* | 504 | 35 | T | C | 0.40 | ACT→ACC | T→T | synonymous |
|  |  | 506 | 37 | C | U | 0.43 | CCA→CUA | P→L | non- |
|  |  | 550 | 44 | T | C | 0.48 | TTA→CTA | L→L | synonymous |
|  |  | 551 | 40 | T | C | 0.50 | TTA→TCA | L→S | non- |
|  |  | 565 | 47 | C | A | 0.51 | CGA→AGA | R→R | synonymous |
|  | *cemA* | 671 | 21 | A | C | 0.19 | TAT→TCT | Y→S | non- |
|  | *ndhD* | 46 | 107 | T | C | 0.79 | TTT→CTT | F→L | non- |
|  |  | 878 | 35 | C | U | 0.97 | TCA→TUA | S→L | non- |
|  | *ndhF* | 62 | 20 | C | U | 0.60 | TCA→TUA | S→L | non- |
|  | *ndhK* | 128 | 165 | C | U | 1.00 | CCA→CUA | P→L | non- |
|  | *petD* | 390 | 20 | A | G | 0.10 | ACA→ACG | T→T | synonymous |
|  | *psaA* | 1713 | 207 | C | U | 0.91 | TTC→TTU | F→F | synonymous |
|  |  | 1723 | 201 | G | U | 0.91 | GGG→UGG | G→W | non- |
|  |  | 1739 | 212 | G | A | 0.91 | GGA→GAA | G→E | non- |
|  |  | 1743 | 228 | A | G | 0.92 | ACA→ACG | T→T | synonymous |
|  |  | 1758 | 230 | C | A | 0.28 | GCC→GCA | A→A | synonymous |
|  |  | 1777 | 207 | G | U | 0.91 | GGT→UGT | G→C | non- |
|  | *psbC* | 798 | 20 | G | A | 0.20 | TGG→TGA | W→* | non- |
|  | *psbN* | 17 | 20 | T | A | 0.60 | TTA→TAA | L→* | non- |
|  |  | 29 | 24 | C | U | 0.25 | TCC→TUC | S→F | non- |
|  |  | 30 | 26 | C | U | 0.23 | TCC→TCU | S→S | synonymous |
|  |  | 39 | 29 | T | G | 0.69 | GGT→GGG | G→G | synonymous |
|  |  | 51 | 28 | C | A | 0.71 | AGC→AGA | S→R | non- |
|  |  | 56 | 30 | C | A | 0.77 | ACT→AAT | T→N | non- |
|  |  | 81 | 34 | C | U | 0.79 | TTC→TTU | F→F | synonymous |
|  |  | 93 | 47 | T | C | 0.57 | TCT→TCC | S→S | synonymous |
|  | *rpl2* | 15 | 21 | A | G | 0.24 | TTA→TTG | L→L | synonymous |
|  |  | 32 | 29 | C | U | 0.17 | CCG→CUG | P→L | non- |
|  |  | 76 | 46 | C | U | 0.15 | CCA→UCA | P→S | non- |
|  |  | 109 | 46 | T | C | 0.11 | TGT→CGT | C→R | non- |
|  |  | 173 | 53 | G | A | 0.11 | CGC→CAC | R→H | non- |
|  |  | 196 | 51 | C | U | 0.14 | CGA→UGA | R→* | non- |
|  |  | 199 | 54 | C | U | 0.13 | CGG→UGG | R→W | non- |
|  |  | 220 | 49 | G | A | 0.18 | GGT→AGT | G→S | non- |
|  |  | 254 | 51 | G | A | 0.22 | CGA→CAA | R→Q | non- |
|  |  | 262 | 40 | T | C | 0.18 | TAC→CAC | Y→H | non- |
|  |  | 437 | 119 | G | A | 0.82 | CGT→CAT | R→H | non- |
|  |  | 440 | 118 | G | A | 0.81 | GGA→GAA | G→E | non- |
|  |  | 445 | 113 | G | A | 0.77 | GGT→AGT | G→S | non- |
|  |  | 451 | 113 | C | U | 0.77 | CAA→UAA | Q→* | non- |
|  |  | 465 | 119 | A | G | 0.70 | GCA→GCG | A→A | synonymous |
|  |  | 480 | 84 | G | A | 0.68 | GCG→GCA | A→A | synonymous |
|  |  | 481 | 81 | A | G | 0.72 | AAA→GAA | K→E | non- |
|  |  | 490 | 85 | G | U | 0.73 | GCA→UCA | A→S | non- |
|  |  | 491 | 82 | C | G | 0.73 | GCA→GGA | A→G | non- |
|  |  | 498 | 84 | A | G | 0.69 | GAA→GAG | E→E | synonymous |
|  |  | 499 | 79 | G | U | 0.70 | GGT→UGT | G→C | non- |
|  |  | 507 | 77 | G | A | 0.71 | TCG→TCA | S→S | synonymous |
|  | *rps12* | 225 | 40 | A | G | 0.10 | CAA→CAG | Q→Q | synonymous |
|  |  | 228 | 40 | A | G | 0.10 | GAA→GAG | E→E | synonymous |
|  |  | 229 | 37 | C | U | 0.11 | CAT→UAT | H→Y | non- |
|  |  | 236 | 34 | T | A | 0.12 | GTA→GAA | V→E | non- |
|  |  | 266 | 33 | A | G | 0.12 | GAT→GGT | D→G | non- |
|  |  | 270 | 30 | A | G | 0.13 | TTA→TTG | L→L | synonymous |
|  |  | 273 | 27 | C | U | 0.15 | CCC→CCU | P→P | synonymous |
|  |  | 279 | 25 | G | A | 0.16 | GTG→GTA | V→V | synonymous |
|  |  | 287 | 24 | G | A | 0.17 | CGC→CAC | R→H | non- |
|  |  | 295 | 22 | C | U | 0.18 | CGA→UGA | R→* | non- |
|  | *rps14* | 231 | 20 | A | U | 0.10 | CGA→CGU | R→R | synonymous |
|  | *rps16* | 40 | 24 | C | U | 0.88 | CAA→UAA | Q→* | non- |
| **MT** | *matR* | 251 | 52 | T | A | 1.00 | TTT→TAT | F→Y | non- |
|  | *rps14* | 25 | 17 | T | C | 1.00 | TTG→CTG | L→L | synonymous |
|  |  | 45 | 17 | G | U | 1.00 | AAG→AAU | K→N | non- |
